# Hsp70 Oligomerization Is Mediated by an Interaction between the Interdomain Linker and the Substrate-Binding Domain

**DOI:** 10.1371/journal.pone.0067961

**Published:** 2013-06-28

**Authors:** Francesco A. Aprile, Anne Dhulesia, Florian Stengel, Cintia Roodveldt, Justin L. P. Benesch, Paolo Tortora, Carol V. Robinson, Xavier Salvatella, Christopher M. Dobson, Nunilo Cremades

**Affiliations:** 1 Department of Chemistry, University of Cambridge, Cambridge, United Kingdom; 2 Department of Biotechnology and Biosciences, University of Milano-Bicocca, Milan, Italy; 3 Department of Chemistry, Physical and Theoretical Chemistry Laboratory, University of Oxford, Oxford, United Kingdom; 4 CABIMER-Andalusian Center for Molecular Biology and Regenerative Medicine (Consejo Superior de Investigaciones Científicas-University of Seville-UPO-Junta de Andalucia), Seville, Spain; 5 Joint BSC-IRB Research Programme in Computational Biology, Institute for Research in Biomedicine (IRB Barcelona), Barcelona, Spain; 6 Institució Catalana de Recerca i Estudis Avançats (ICREA), Barcelona, Spain; Semmelweis University, Hungary

## Abstract

Oligomerization in the heat shock protein (Hsp) 70 family has been extensively documented both *in vitro* and *in vivo*, although the mechanism, the identity of the specific protein regions involved and the physiological relevance of this process are still unclear. We have studied the oligomeric properties of a series of human Hsp70 variants by means of nanoelectrospray ionization mass spectrometry, optical spectroscopy and quantitative size exclusion chromatography. Our results show that Hsp70 oligomerization takes place through a specific interaction between the interdomain linker of one molecule and the substrate-binding domain of a different molecule, generating dimers and higher-order oligomers. We have found that substrate binding shifts the oligomerization equilibrium towards the accumulation of functional monomeric protein, probably by sequestering the helical lid sub-domain needed to stabilize the chaperone: substrate complex. Taken together, these findings suggest a possible role of chaperone oligomerization as a mechanism for regulating the availability of the active monomeric form of the chaperone and for the control of substrate binding and release.

## Introduction

The 70-kDa heat shock proteins (Hsp70s) are essential members of the cellular chaperone machinery, assisting protein folding, disaggregation and trafficking, and their malfunction has been associated with a wide variety of diseases, including cancer, neuro-degeneration, allograft rejection and infection [1]. Hsp70s are ubiquitously expressed in organisms ranging from *Archaea* to *Homo sapiens* and they are among the most conserved proteins in evolution [2]. This characteristic has allowed conclusions from the studies of different Hsp70 homologues to be extrapolated to the entire Hsp70 family.

Hsp70 proteins consist of two structurally independent domains: a conserved nucleotide-binding domain (NBD) with ATPase activity, which in *H. sapiens* Hsp70 corresponds to residues 1-383, and a more variable C-terminal substrate-binding domain (SBD), corresponding to residues 397-641. Two subdomains can be differentiated within the SBD: a β-sandwich region (residues 397-507) that contains the substrate-binding pocket (substrate-binding subdomain - SBSD), and a 10 kDa subdomain of predominantly α-helical structure (residues 508-641), also named the helical lid subdomain (HLS), which has been suggested to play a key role in regulating the kinetics of substrate binding [3]. Finally, the NBD and the SBD are connected by a hydrophobic linker of 13 residues (384-396) that carries a highly conserved leucine-rich motif. Recent evidence suggests that this linker might play an active role in the allosteric communication between domains and in the recruitment of the co-chaperone Hsp40 [4-7].

The HLS is comprised of five helices (named helices A-E) and an intrinsically disordered region corresponding to the last 26 residues, and has been proposed to act as a lid over the β-sandwich subdomain upon substrate binding [8,9]. The entire HLS region has been shown to be highly mobile and this dynamic behaviour has been found to be a pre-requisite for the chaperone to be able to accommodate a broad spectrum of client polypeptides into the binding pocket [10,11]. Despite these observations, the details of the substrate binding mechanism remain unclear and the nature of possible interactions between substrate proteins and the HLS is not yet fully resolved. Moreover, the HLS has been shown to be essential in stabilizing Hsp70-substrate complexes [12-14], and could in addition induce conformational changes in the bound substrates and hence play an important role in chaperone function [10]. In agreement with this idea, a lidless variant of DnaK has been reported to be unable to stimulate the refolding of chemically denatured firefly luciferase, suggesting that the HLS may indeed play an important part in the refolding of some substrates [3].

Oligomerization through the formation of dimers and higher-order oligomers has been reported both *in vitro* and *in vivo* for a substantial number of Hsp70 family members, including constitutive rat Hsc70 [15-19], mouse [20], bovine [21], plant [22] and human [23,24] inducible Hsp70, murine [25], bovine [26] and hamster [27-29] endoplasmic reticulum resident BiP/Grp78, and the bacterial homologue DnaK [30,31]; these observations have led to suggestions that the oligomerization process could be a common feature of the Hsp70 family. It has been shown, for example, that up to 40-50% of the molecules of the chaperone BiP/Grp78 exist as oligomers both *in vitro*, at ca. 5 µM of protein [28], and in mammalian cells [29], and it has been suggested that the inter-conversion between oligomeric and monomeric species may be related to the chaperone function of the protein [16,23,25-29]; similar results have been obtained for other Hsp70 homologues, where the oligomerization process has been shown to be reversible [16,19-24]. Differences in the propensity to form oligomers at low micromolar concentrations have been found to occur under different conditions and to vary between different Hsp70 variants. Specifically, it has been observed that the nucleotide free- and ADP-bound forms of the chaperone are prone to self-oligomerization *in vitro* in a concentration and temperature dependent manner, processes that are reversed by the addition of ATP, substrate binding, and the presence of some co-chaperones [15,16,30]. The bacterial homologue, DnaK, has been found to form oligomers *in vitro*, with the degree of oligomerization varying with the protein concentration; approximately 20% of the protein is in an oligomeric state at 0.5 mg/ml of protein (ca. 7 µM), rising to 70% at 16 mg/ml (ca. 230 µM) [30]. The formation of oligomers of DnaK has also been found to be reversed by the addition of ATP and, interestingly, similar proportions of monomers, dimers and other small but well defined oligomers were observed in *Escherichia coli* cellular lysates [31], demonstrating that the oligomerization observed *in vitro* also takes place *in vivo*. When these DnaK oligomers were chemically cross-linked, and then isolated from the cellular environment, they were found to retain some ATPase activity and to bind model substrates, albeit with a reduced ability to induce refolding [31]. The general relevance of these oligomeric species to the chaperone function is still unclear, although several studies have suggested an important role for the oligomerization process in the chaperone cycle, primarily as a regulatory mechanism for the availability of the active monomeric form of the chaperone [16,23].

Despite the extensive studies reporting the formation of Hsp70 oligomers both *in vitro* and *in vivo*, the mechanism by which these oligomeric species are formed and the nature of the interactions between the oligomerization interfaces are unclear. Different locations for the interactions leading to oligomerization have been proposed, including regions between residues 385-540 within the β-subdomain of the SBD, in bovine Hsc70 [17], as well as regions of the HLS of the SBD, such as the segment between residues 554-646 in rat Hsc70 [32] or even regions spanning both subdomains (residues 382-561 of human Hsp70 [24]). With the aim of shedding more light on the specific protein regions involved in Hsp70 oligomerization and its mechanism, as well as on the possible role that this process could play in the chaperone functional cycle, we have characterized the oligomerization propensity, as well as its impact on the substrate binding of a series of human Hsp70 constructs by means of nanoelectrospray ionization mass spectrometry (nESI-MS), analytical size exclusion chromatography (SEC) and fluorescence and circular dichroism (CD) spectroscopies.

## Materials and Methods

### Protein expression and purification

Recombinant N-hexa-His-tagged Hsp70 (human Hsp70 1A, GenBank ID: NP005336) and all the truncated SBD variants discussed here were overexpressed from pET-28b vector (Merck KGaA, Darmstadt, Germany) in *E. coli* BL21 (DE3) Gold Strain (Agilent Technologies, Santa Clara, USA) and purified by affinity chromatography as previously described [33]. The monomeric fraction was in each case isolated by SEC using a Superdex 26/60 G75 column (GE Healthcare LifeSciences, Little Chalfont, U.K.). Thrombin cleavage efficiency, estimated by mass spectrometric analysis, was greater than 99% for all the variants and the protein purity, as determined by SDS–PAGE, exceeded 95%. Solutions of the purified proteins were then divided into aliquots, flash-frozen in liquid nitrogen and stored at -80 ^°^C; each protein aliquot was thawed only once before use. Protein concentrations were determined by absorbance measurements at 280 nm using theoretical extinction coefficients calculated with Expasy ProtParam [34].

### Circular dichroism spectroscopy

Far-UV CD spectra for all protein variants were recorded using a Jasco *J-810* spectropolarimeter equipped with a Peltier holder, using a 0.1 cm path length cuvette. Typically, samples contained 20 µM protein in 7 mM Tris buffer at pH 7.4, containing 170 mM KCl and 5 mM MgCl_2_. The far-UV CD spectra of all the variants in their native, thermally denatured and refolded states were recorded from 198 to 250 nm, and the spectrum of the buffer was systematically subtracted from the spectra of all protein samples. The secondary structure content was estimated from the far-UV CD spectrum for each protein variant using K2D software [35].

Protein structural stability was analyzed by monitoring the CD signal at 222 nm upon thermal denaturation. Thermal runs were performed from 5 to 90 °C at a rate of 1 °C. min^-1^. Data points were acquired every 0.1 °C with a response time of 2 s and a bandwidth of 1 mm. After thermal denaturation, each protein sample was cooled to the starting temperature, and a second denaturation curve recorded in order to analyse the degree of reversibility of the denaturation process. Analysis of the thermal unfolding curves was performed assuming a three-state unfolding model [36].

### Fluorescence spectroscopy

Steady-state tryptophan fluorescence emission spectra of native, thermally denatured and refolded proteins were recorded from 300 to 400 nm, selectively exciting the only tryptophan residue in the SBD (W580) at 295 nm, using a Cary Eclipse spectrofluorimeter (Varian, Palo Alto, CA, USA). Excitation and emission band-passes were set to 5 nm each.

### Analytical size exclusion chromatography

Analytical SEC was performed at 4 °C using a Superdex 75 10/300 GL column (GE Healthcare LifeSciences, Little Chalfont, U.K.), which had previously been calibrated using a mixture of standard proteins (Gel Filtration LMW Calibration Kit; GE Healthcare LifeSciences, Little Chalfont, U.K.). Typically, samples of the various constructs contained 70 µM of protein in 50 mM Tris buffer at pH 7.4, with 150 mM KCl, 5 mM MgCl_2_ and were incubated for 15 min (unless otherwise specified) at room temperature before being loaded onto the column for analysis. To evaluate the reversibility of the oligomerization process, the different elution peaks obtained for each protein variant were subsequently analyzed after concentrating the different fractions using AmiconUltra-0.5, Ultracel-3 Membrane, 3 kDa (Millipore, Bedford, MA, USA). A similar analysis was also performed at 6 µM concentration for all the protein variants to allow for a direct comparison between the results obtained by SEC and MS experiments.

The influence of substrate binding on the oligomerization properties of all the variants was examined by using the well-characterized Hsp70 substrate, the NR peptide (NRLLLTG, Genemed Synthesis Inc., San Antonio, TX, USA). A 14-fold excess of the peptide was added in each case to a fresh protein sample, incubated for 1 h at room temperature, unless otherwise specified, and then loaded onto the Superdex 75 10/300 GL column for analysis. The relative quantities of the different protein species were estimated by analysing the chromatographic profile using a Gaussian function for each elution peak (OriginPro 8, Originlab, Northampton, MA, USA) and measuring the relative area under each peak.

### Mass spectrometry

Prior to MS analysis, samples were buffer exchanged into 150 mM ammonium acetate, pH 7.4, using Micro Bio-spin 6 columns (Bio-Rad, Digilab Division, Cambridge, MA, USA). For all the experiments protein concentrations ranging from 6 to 18 µM were used. In the case of the substrate binding experiments, proteins were analyzed in the presence of a 1: 1 molar ratio of the NR peptide; this substrate concentration, lower than in the SEC experiments, was chosen to ensure adequate resolution of the various charge state series, which is critical for unambiguous assignment of substrate-bound states. Due to suppression effects during nESI ionization, a relatively limited set of molar ratios could be investigated by in this way.

nESI-MS spectra were obtained according to a previously published protocol [37] on an orthogonal time-of-flight mass spectrometer with a Z-spray source (LCT, Waters, Manchester, UK) in positive ion mode using the following parameters: capillary voltage, 1500 V; sample cone voltage, 120 V (unless otherwise specified); extraction cone voltage, 1V; and ion transfer stage pressure, 8.8 mbar. Spectra were calibrated externally using a 33 mg/ml solution of cesium iodide. Data were acquired and processed with MassLynx software (Waters, Manchester, UK), and are shown with minimal smoothing and without background subtraction. The relative abundances of the different oligomeric states were estimated from the intensities of the most abundant charge states for each construct and are expressed as a percentage of the total intensity.

**Figure 1:**
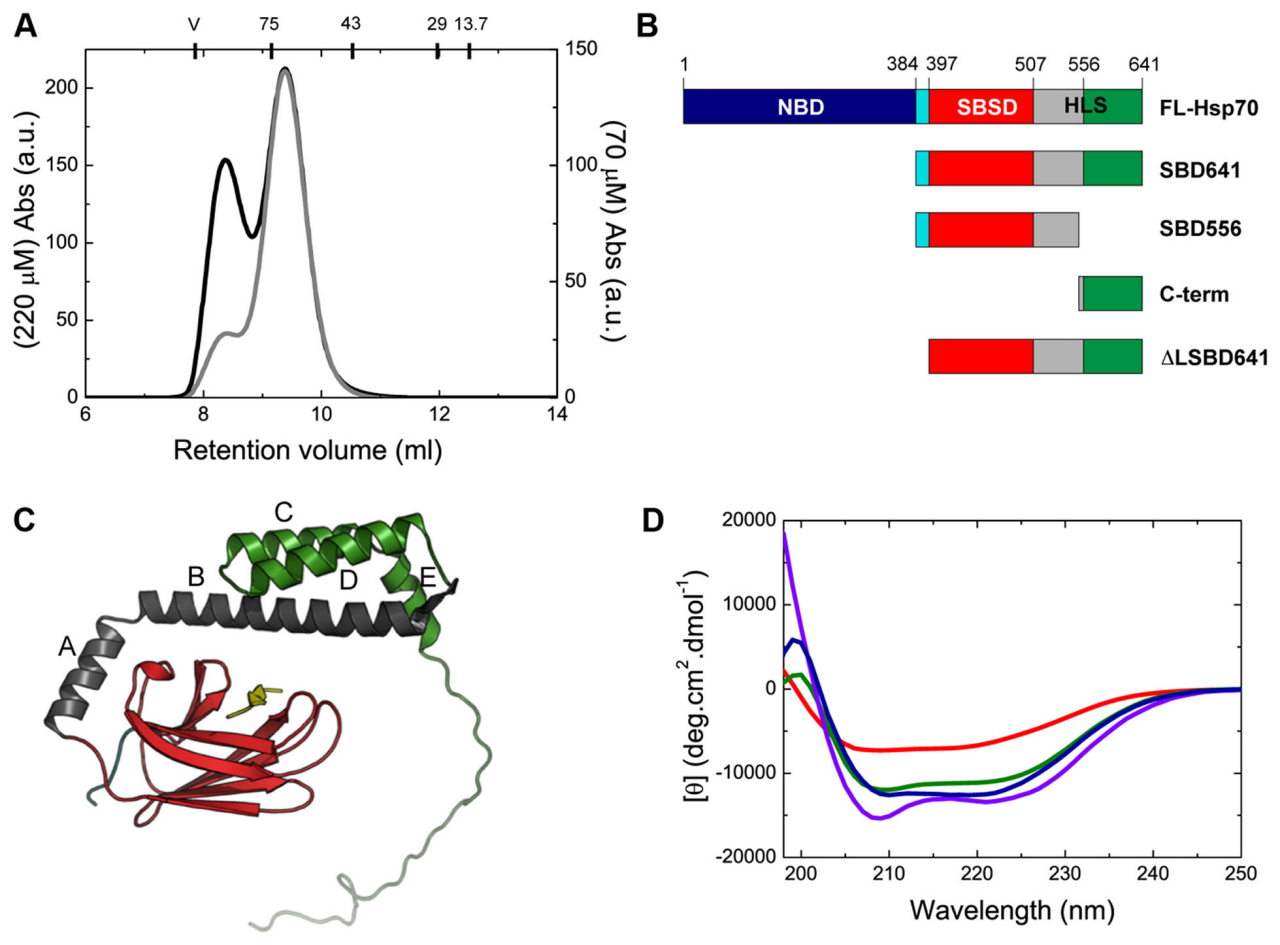
The different human Hsp70 variants maintain native-like structure. (A) Analytical SEC analysis of FL-Hsp70 at protein concentrations of 220 µM (black line) and 70 µM (grey line). At the top of the chromatogram the molecular weights of the following standard proteins used to calibrate the column are reported at the position which each elutes: conalbumin (75 kDa), ovalbumin (43 kDa), carbonic anhydrase (29 kDa) and ribonuclease A (13.7 kDa). The void volume (V) of the column (7.8 ml) was determined using blue dextran. (B) Schematic representation of the engineered human Hsp70 variants used in this study, showing the different functional domains for each variant: the nucleotide-binding domain, NBD, shown in dark blue, the interdomain linker shown in light blue and the substrate binding domain, SBD, which is composed of the substrate-binding subdomain, SBSD, shown in red, and the helical lid subdomain, HLS, with helices A-B shown in grey and helices C-E and the C-terminal tail shown in green (see text for a detailed description of the truncated variants). (C) Representation of the different truncated constructs on the crystal structure of the DnaK SBD in complex with the NR peptide substrate, represented in yellow (adapted from PDB ID: 1DKX [8]). (D) Far-UV CD spectra of the native FL-Hsp70 (blue), SBD641 (green), SBD556 (red) and C-term (violet) variants (see Table S1 in File S1 for a detailed analysis of the secondary structure content of each chaperone variant).

Complementary “denatured” spectra under acidic conditions were obtained by adding AG501-X8 resins (Bio-Rad, Digilab Division, Cambridge, MA, USA) to the samples. In general, the charge states of proteins shift towards high charge ranges (lower *m/z*) when examined under denaturing conditions, indicative of a higher degree of unfolding [38]. Intriguingly, the MS of the 'C-term' variant is very similar under native and denaturing conditions (Figure S1). In Tris buffer at physiological pH (7.4) and 25 °C, this variant has a high content of helical structure according to its far-UV CD spectrum (Figure 1D), consistent with that expected from the crystal structures of homologues proteins (Table S1 in File S1). It therefore appears to maintain a high degree of secondary structure even under acidic conditions, resulting in a similar mass spectrum under both sets of conditions.

### NR peptide binding to Hsp70 variants

The interaction between the different Hsp70 variants and the NR peptide was studied in 50 mM Tris buffer at pH 7.4, with 150 mM KCl, and 5 mM MgCl_2_ using fluorescence spectroscopy by titrating different concentrations of chaperone into a solution containing 2 µM dansyl-NR peptide (N-terminally labeled; Genemed Synthesis Inc., San Antonio, TX, USA) after incubating the mixtures for 1 h at 25°C. The concentration of dansyl-NR peptide was calculated using an extinction coefficient at 327 nm of 4300 M^-1^.cm^-1^ [39]. The fluorescence emission spectrum of each sample was recorded from 400 to 630 nm after excitation at 330 nm, and the data reported correspond to the average intensity at the maximum emission wavelength of two different samples for which ten spectra were recorded. The increase in fluorescence signal was plotted as a function of chaperone concentration (Figure S2), and analyzed assuming single-site binding using the following equation:

$$\Delta F=\Delta F_{\max}\Theta_{L}=\frac{\Delta F_{\max}}{2L_{T}}\left[\left(P_{T}+L_{T}+K_{d}\right)-\sqrt{{\left(P_{T}+L_{T}+K_{d}\right)}^{2}-4P_{T}L_{T}}\right]$$ (1)

where Θ_L_ is the fraction of bound ligand, Δ*F* is the increase in fluorescence intensity observed at a given concentration of Hsp70 variant, Δ*F*_max_ is the increase in fluorescence at saturation, *L*_*T*_ and *P*_*T*_ are the total ligand and protein concentration respectively, and *K*_*d*_ is the apparent dissociation constant of the complex. The fraction of bound ligand was plotted as a function of protein concentration in order to compare affinities between the different chaperone variants.

## Results

In the present work we have investigated human Hsp70 in order to probe the oligomerization process in a representative member of the Hsp70 protein family. We find, as previously reported [24], that human Hsp70 is able to form oligomers *in vitro* in a concentration-dependent manner under near-physiological conditions, in agreement with results from other members of the family [30]. At a protein concentration of 220 µM, 35% of full-length Hsp70 (FL-Hsp70) exists as oligomeric species, although this percentage drops to ca. 10% at 70 µM protein (Figure 1A). With the aim of shedding light on the details of the mechanism of Hsp70 oligomerization, we generated a series of differently truncated variants of human Hsp70, carefully designed to preserve the native structural topology of the full-length protein while displaying different oligomerization propensities.

### Design of truncated variants of human Hsp70

Different regions within the SBD of various Hsp70 family members have been suggested to be involved in chaperone oligomerization, located either in the SBSD [16,17] or in the C-terminal segment of the HLS of the SBD [24,32,40]. Based on this information, we generated a set of truncated SBD variants of human Hsp70 (Figure 1B and 1C): SBD641 (384-641), corresponding to the full-length SBD; SBD556 (384-556), which lacks helices C, D and E and the C-terminal disordered region; ΔLSBD641 (394-641), corresponding to SBD641 without the first ten residues of the interdomain linker (SENVQDLLLL); and the 'C-term' variant (540-641), consisting of the C-terminal segment of the helix B, helices C-E and the disordered C-terminal region of the HLS. The truncation points were designed on the basis of published structures of the homologous proteins (PDB IDs: 1DKX, 7HSC, 1UD0, 1HJO) (Figure 1C). In the case of the SBD556 variant we also incorporated the L542Y mutation in order to prevent self-binding of the leucine residue at position 542 to the substrate-binding pocket, as previously reported for a similar truncated variant of DnaK [41]. The Dnak truncated variant was shown to have an extremely low affinity for substrates when compared with the full-length protein, but the affinity was recovered when the leucine residues at position 542 and 543 were substituted for tyrosine and glutamate residues, respectively [41]. In our SBD556 variant, only the leucine residue at position 542 needed to be substituted as this variant already contains a glutamate residue at position 543. Self-binding by the human SBD556 variant in the present study was experimentally excluded by its ability to bind substrates with an affinity of the same order of magnitude as the full-length protein.

The structural integrity of all the variants, including FL-Hsp70, was studied at 25 °C using far-UV CD (Figure 1D). The secondary structure content for all the variants, derived by analysis of their far-UV CD spectra, is in excellent agreement with that predicted from the available X-ray structures (Table S1 in File S1), indicating that the different truncated variants are able to fold and generate the native-like structure of the two domains without significant differences in structure relative to the full-length protein. The structural and thermodynamic stability of the truncated variants was assessed by thermal unfolding experiments, where all the variants showed high stability and cooperativity (see later in the text).

### Analysis of human Hsp70 variants using nanoelectrospray ionization mass spectrometry and analytical size exclusion chromatography

**Figure 2:**
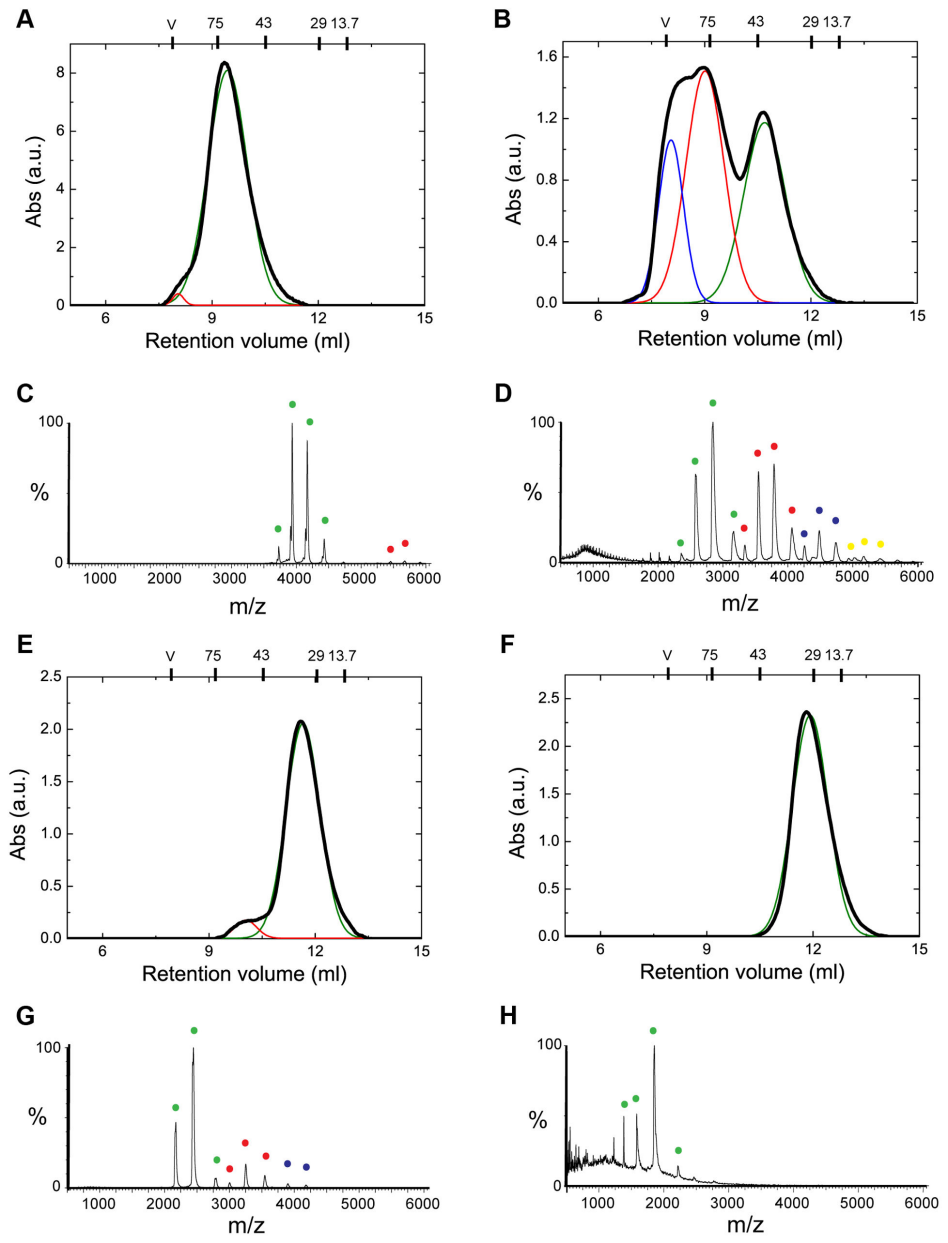
The different protein variants have different propensities to oligomerize. Analytical SEC and nESI mass spectra of the different protein constructs measured at the same protein concentration (6 µM): FL-Hsp70 (A, C), SBD641 (B, D), SBD556 (E, G) and C-term (F, H) variants. The SEC elution profiles were fitted to multi-peak Gaussian functions to evaluate the relative fractions of monomeric and oligomeric protein species. The molecular masses of the standard proteins used to calibrate the SEC column are reported at the top of the chromatogram. The elution peaks in SEC and the peaks in MS (Table S2 in File S1) corresponding to monomeric protein are coloured in green for all the variants, while those for dimers are in red, trimers in blue and tetramers in yellow. Note that comparing visually the SEC and MS data quantitatively is difficult as the relative peak heights in the mass spectra report on molecular abundance, which is independent of oligomeric state, while the SEC data depend on molecular absorbance.

nESI-MS is a highly effective technique for quantifying the relative abundances of the different components of heterogeneous and polydispersed molecular chaperone molecule [42-44]. We have capitalized on this ability to assess the relative proportions of monomers and oligomers in solution of the different protein variants that we generated in this study (Figures 2C-D and 2G-H, Table 1 and Table S2 in File S1). We first compared the mass spectra of each variant under native (Figure 2) and denaturing (Figure S1) solution conditions [37]. These data indicate that some of the truncated protein variants have a clear tendency to oligomerize, even at the low (μM) concentrations used for the MS experiments, and reveal important differences in this regard between the different variants. Specifically, the SBD641 variant shows the highest degree of oligomerization, with the majority of the protein (54% in total) populating an oligomeric state (40% dimers, 12% trimers and 2% tetramers). SBD556, by contrast, exhibits a considerably smaller proportion of oligomers under the same conditions (ca. 18% in total). This strongly supports the hypothesis that the C-terminal segment of the HLS is involved in the formation of oligomers, as it was previously proposed. According to the MS analysis, the 'C-term' variant is monomeric under the conditions used (see Materials and Methods), and the data for FL-Hsp70 reveal only a very small proportion of dimers (ca. 2% at a protein concentration of 6 µM), although the relative populations of oligomeric forms of the protein increases significantly at higher protein concentrations (Figure 1A).

**Table 1:** Relative populations of oligomeric forms of Hsp70 variants^a^.

**Protein Variant**	**6-15 µM protein**								**70 µM protein**				
	**MS (%)**					**SEC (%)**				**SEC (%)**			
	**Mon.**	**Dim.**	**Trim.**	**Total Oligom.**	**Mon.**	**Dim.**	**Total Oligom.**	**Mon.**	**Dim.**	**Total Oligom.**
**FL-Hsp70**	98	2	0	2	99	1	1	93	7	7
**SBD641**	46	40	10	54	37	44	63	23	55	77
**∆LSBD641**	92	8	0	8	91	9	9	86	14	14
**SBD556**	82	16	2	18	97	3	3	93	7	7
**C-term** ^b^	100	0	0	0	100	0	0	100	0	0

a Estimated experimental error is 5% for MS and 3.5% for SEC (standard deviation of 3-4 independent experiments).

b The C-term variant contains residues 540-641.

The data obtained by means of MS were then complemented with analytical SEC measurements performed in parallel at the same protein concentrations (Figures 2A-B and 2E-F). The agreement between the two techniques is very good (Figure 2 and Table 1); this observation suggests there is no significant dissociation of the various oligomers during the SEC experiments (note that these experiments were performed at 4 °C, which presumably considerably slows down the dissociation of complexes) and, therefore, that the populations obtained by this technique represent good estimates of the populations contained in the initial samples. This assumption was further confirmed by using dynamic light scattering measurements (Figure S3B).

SBD641 can be seen to be largely oligomeric at a concentration of 6 µM. The rest of the protein variants are largely monomeric with only SBD556 revealing a significant population of oligomers at this low protein concentration (Table 1). The 'C-term' variant was found to elute as a single peak, in agreement with the MS results where only monomers are detected. The Stokes radius derived from the retention volume (21.4 Å) is consistent with that estimated from the crystal structure of the same truncated variant from *Caenorhabditis elegans* (22.5 Å), with which it shares 96% sequence similarity [40]. This result, however, contrasts with other studies where dimers of a very similar truncated variant of rat Hsc70 have been reported in solution and also observed in the crystal structure [32]. To show further that, under our experimental conditions, this protein variant is monomeric, samples at concentrations ranging from 1 to 125 µM were analyzed by analytical SEC and a single elution peak, centred at exactly the same retention volume (11.8 ml) was obtained at all concentrations (Figure S4). For the other protein variants including FL-Hsp70, however, a significant increase in the fraction of oligomeric species was observed when the protein concentration was increased from 6 to 70 µM (Table 1 and Figure S5), a result that also indicates that the interactions leading to oligomerization in Hsp70 have affinities in the μM range.

Comparing the original SEC chromatograms with those obtained by individually re-loading the monomeric and oligomeric fractions in the same chromatography column (see Materials and Methods), enabled us to assess the reversibility of the oligomerization process. Both isolated monomeric and oligomeric fractions of FL-Hsp70 and all truncated variants (except the 'C-term' variant) examined in this manner gave rise to chromatograms with two elution peaks, corresponding closely to the elution volumes of the monomeric and oligomeric species originally obtained (Figure S6), indicating that the self-assembly process for all variants corresponds to reversible equilibria between monomeric and oligomeric species.

### Influence of the interdomain linker on oligomerization

The large differences between the fractions of oligomeric species formed with the SBD641 and SBD556 variants (Table 1), which differ only in the presence or absence of 85 residues of the C-terminal segment of the HLS (Figure 1B and 1C), suggests that this C-terminal region of the protein is able to influence the oligomerization of the protein, a finding in accord with previous suggestions [20,24,32]. In addition, our results show that the C-terminal region is unable to dimerize in isolation, at least under our experimental conditions. These results, together with the fact that a direct interaction of the C-terminal region of one molecule with that from a second molecule would not allow the formation of higher-order oligomers such as the trimers and tetramers observed in this work as well as in previous studies on homologues Hsp70 proteins [16,17,23,31], led us to search for a different region of the protein through which the SBD of the protein is able to interact to form oligomeric species.

**Figure 3:**
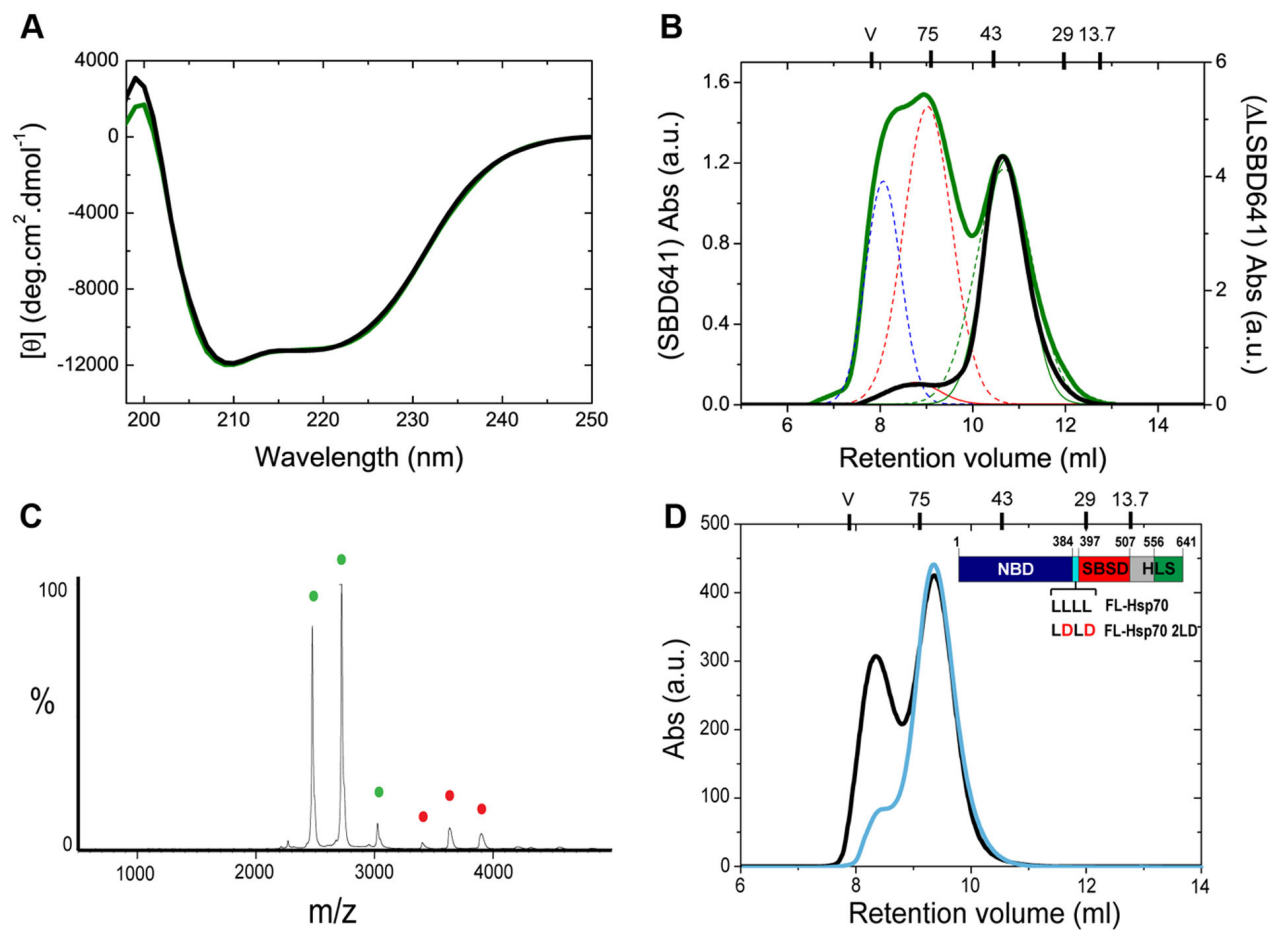
The interdomain linker is essential for chaperone oligomerization. (A) Far-UV CD spectrum of native ∆LSBD641 (black) compared to that of the SBD641 variant (green). (B) Comparison of the SEC chromatograms obtained for SBD641 (in green) and ΔLSBD641 (in black) at a protein concentration of 6 µM. (C) nESI MS analysis of ΔLSBD641 (monomer in green, dimer in red). (D) Comparison of the SEC chromatograms obtained for FL-Hsp70 (in black) and FL-Hsp70 2LD (in cyan) at a protein concentration of 220 µM. The molecular masses of the standard proteins are again reported at the top of the chromatogram.

The NBD and SBD of Hsp70 are connected by a hydrophobic leucine-rich linker of 13 residues, which is included in all the truncated SBD protein variants containing the β-sheet subdomain that we initially examined. To explore the possibility of an interaction between the C-terminal tract of the SBD and the interdomain linker, therefore, we generated a modified SBD641 variant, denoted ΔLSBD641, in which the interdomain linker is absent. This variant is structurally and thermodynamically identical to the original SBD641, as assessed by MS in denaturing conditions, far-UV CD and thermal unfolding (Figure 3A, Figure S7, Figure S8A and Table S1 and Table S3 in File S1), but its propensity to oligomerize is dramatically reduced; >90% of all protein molecules detected by both analytical SEC and MS are monomeric (Figure 3B and 3C, Figure S5 and Table 1), in contrast to the original SBD641 variant (Figure 2B and 2D, Figure S5E and Table 1) where the oligomeric species represent >50% of the overall population at the same protein concentration.

In order to investigate further the role of the interdomain linker in chaperone oligomerization, we generated a FL-Hsp70 variant (FL-Hsp70 2LD), in which we specifically disrupted the conserved leucine-rich tract of the interdomain linker by substituting two aspartic residues for Leu395 and Leu397 (Figure 3D). When we analyzed the oligomerization propensity of this new protein variant in relation to the wild type full-length protein, we found that the two leucine substitutions exert a dramatic effect on oligomer formation (Figure 3D), although they do not detectably affect the structural properties of the protein (Figure S9). Indeed, FL-Hsp70 2LD at a protein concentration of 220 µM was found to exist essentially as monomeric protein (less than 9% of the protein was in an oligomeric state), while for wild type FL-Hsp70, 35% of the protein was found to form oligomers at the same protein concentration (Figure 3D). This result demonstrates that the interdomain linker is one of the protein interfaces involved in the oligomerization of Hsp70 truncated variants as well as in the full length protein. At the same time, it validates the use of our protein constructs to gain a general understanding of the self-association mechanism.

These findings provide significant insights into the oligomerization process of Hsp70 as they reveal that two distinct regions of the protein, located in different folding and functional domains, are involved in the process: the SBD, and the leucine-rich motif of the interdomain linker between the SBD and the NBD. The different oligomerization propensities of SBD641 and FL-Hsp70, which have structurally identical SBDs, can be explained by different accessibilities of the interdomain linker to interact with the SBD of another chaperone molecule. This conclusion is suggested by analysis of the crystal structures of Hsp70 homologues [8,45] that reveals a 25% reduction in the solvent accessible surface area (SASA) of the interdomain linker in bovine FL-Hsc70 [45] when compared to the DnaK SBD [8]. The different accessibilities of the interdomain linker in SBD641 and FL-Hsp70 further suggests that this region represents the primary binding site of the SBD responsible for oligomerization, and that its solvent accessibility is a major factor in controlling chaperone oligomerization. The fact that the removal of the first ten residues of the interdomain linker in ΔLSBD641 (or the substitution of two leucine residues of the linker in FL-Hsp70 2LD) does not abolish completely the formation of oligomers (Table 1) suggests that this oligomerization interface is relatively large and is likely to involve a adjacent ensemble of residues of the N-terminal part of the SBD.

### The interaction between the interdomain linker and the SBD is lost following irreversible thermal misfolding of helices C–D

Recent evidence has shown that the distal part of the HLS, particularly helix C, represents a key element for the allosteric regulation of the chaperone activity in several Hsp70 proteins [20,32]. Furthermore, self-oligomerization of Hsp70 has been reported to be influenced *in vitro* by temperature [23,31]. In order to assess whether or not this part of the SBD could play an important role in the oligomerization process of the chaperone, we have analyzed the thermal structural stability of each truncated variant of Hsp70 in relation to its oligomerization propensity.

**Figure 4:**
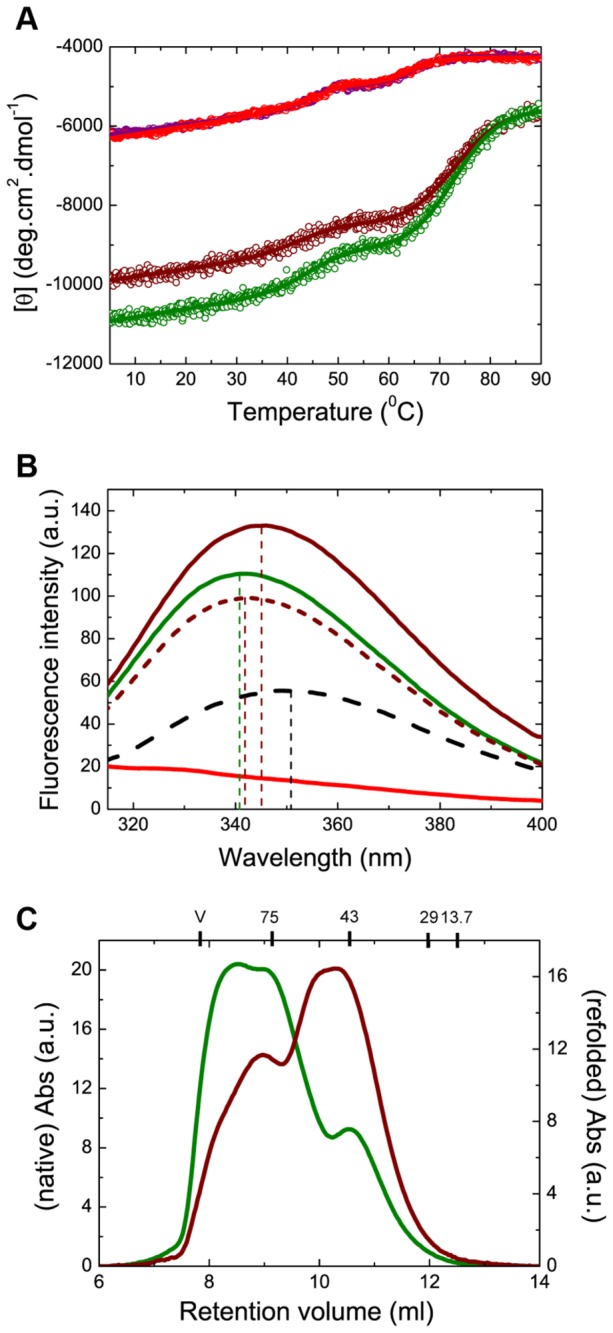
Misfolding of HLS prevents oligomerization. (A) Far-UV CD data for the thermally-induced denaturation of the native and refolded protein SBD556 (red and purple circles, respectively) and SBD641 (green and brown, respectively); a three-state denaturation model was used to analyse the experimental data (continuous lines; see Materials and Methods and Table S3 in File S1). (B) Tryptophan (W580) fluorescence spectra of SBD641 in their native (green line), thermally denatured (black dashed line) and refolded (brown continuous line) states; the spectrum of SBD556 in its native state is shown as a negative control (red continuous line). The calculated spectrum of SBD641, corresponding to 80% of the protein molecules in the native structure and 20% of the molecules in the thermally denatured state, assuming that the total fluorescence signal at a given wavelength is a linear combination of the fluorescence signal for each populated state (brown dashed lines), is also shown for comparison with the spectrum of the protein in its refolded state. (C) Analytical SEC performed on native and refolded (after thermal denaturation of the protein) states of SBD641 (green and brown lines, respectively). The molecular weights of the standard proteins used to calibrate the column are reported at the top of the chromatogram.

We first performed thermal denaturation experiments that involved monitoring the secondary structure content of each protein variant as a function of temperature (Figure 4A and Figures S8A and S8C). The thermal unfolding of the SBD556 and SBD641 variants was found to be very similar when monitored by far-UV CD, with two well-resolved transitions that we have analyzed using a classical three-state model. The first transition is essentially identical for both variants, with the temperature of the mid-point of the transition (*T*_*m1*_) being 45.8 ± 1.0 °C; we can attribute this transition to the denaturation of mainly the β-sheet subdomain of the SBD, residues 397 to 508 [46]. The second transition at higher temperatures can be associated with the denaturation of the HLS, which corresponds to helices A and B (residues 509 to 556) in the case of SBD556 (*T*_*m2*_ = 64.5 ± 0.2 °C), and to the complete HLS (residues 509 to 641) in SBD641 (*T*_*m2*_ = 73.1 ± 0.2 °C; see Table S3 in File S1). In addition, the degree of reversibility of the thermal unfolding process was found to differ between the two constructs; while the unfolding of SBD556 is fully reversible, that of SBD641 is not, indicating that the lack of reversibility is associated with the C-terminal segment of the HLS (helices C-E and the disordered region of the protein that follows). The truncated 'C-term' variant, however, was found not only to adopt a thermodynamically stable structure (Table S3 in File S1), but also to denature both cooperatively and reversibly (Figure S8C).

To characterize further the origin of the irreversibility of the thermal unfolding of the C-terminal segment of the HLS in the SBD641 construct, we assessed the solvent accessibility of the only tryptophan residue in the SBD (W580), which is located at the end of helix C in the HLS, in both the native, thermally unfolded and refolded states of SBD641. The wavelength at the fluorescence emission maximum of the native state of the protein is located at 340 nm and it is red-shifted to 350 nm when the protein is thermally denatured, an observation that is characteristic of a tryptophan residue undergoing a transition from a highly hydrophobic environment in a native protein to a hydrophilic, solvent exposed, environment in the denatured state. The spectrum of the refolded protein has its maximum intensity at 345 nm, a wavelength intermediate between that found for the native and denatured states (Figure 4B). However, the intermediate degree of solvent exposure of W580 in the refolded conformation does not agree with either the degree of irreversibility found for the overall protein thermal denaturation (ca. 20%, see Figure 4A), or the tendency of the variant to oligomerize, as exactly the same behaviour was found to occur in the ∆LSBD641 variant (Figures S8A and S8B). These findings indicate that the region where W580 is located (helix C) does not exist in either the native or the fully-denatured conformations following thermal denaturation; instead, it adopts an alternative conformation where the region around helix C is partially solvent exposed. When this type of analysis was performed on the 'C-term' variant, a red-shift in the maximum wavelength of the fluorescence emission spectrum from the native to the refolded form of the protein was not observed (Figure S8D), in agreement with the high reversibility of the thermal unfolding of this variant. This finding suggests that the thermal irreversibility of the C-terminal region of the HLS results from non-native interactions of this region with the rest of the protein that are formed during the refolding process.

**Figure 5:**
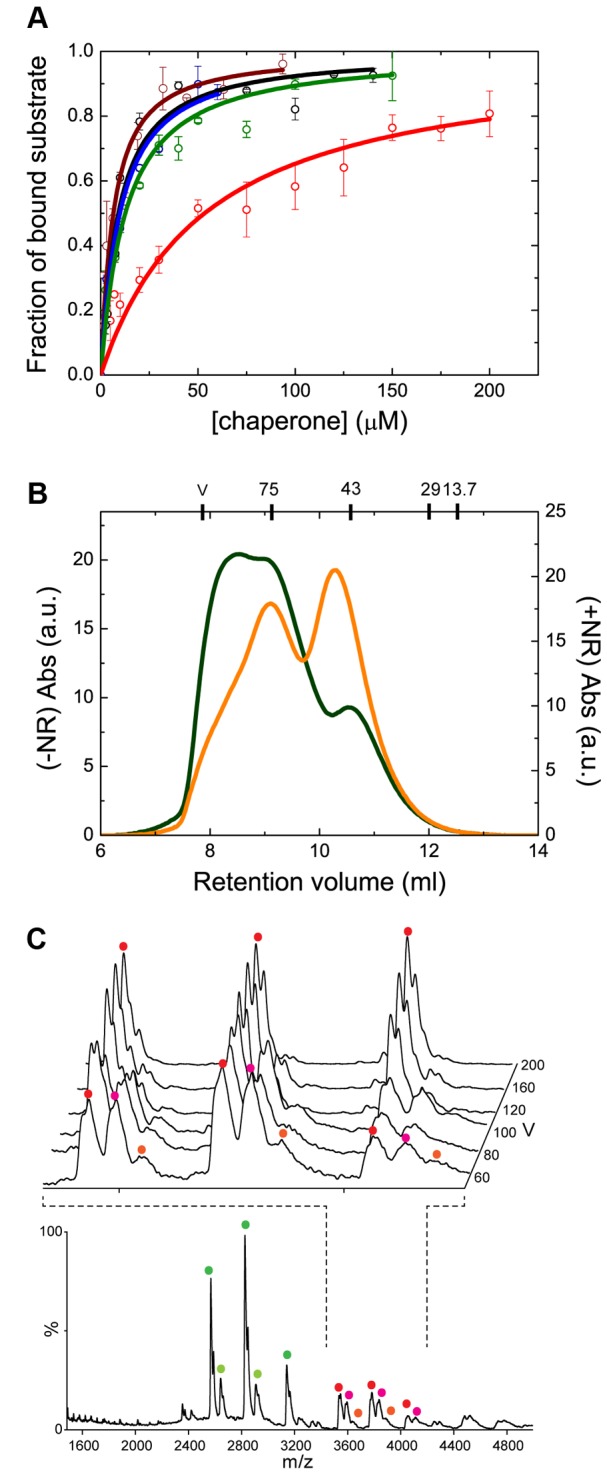
Substrate binding affects the oligomerization equilibrium. (A) Fluorescence titration of the binding of the NR peptide to the different Hsp70 constructs (showing the fraction of bound substrate as a function of chaperone concentration): SBD556 (red circles), native (green circles) and refolded (brown circles) SBD641, ΔLSBD641 (black circles), and FL-Hsp70 (blue circles). The continuous lines represent the best fits of the data to a single binding site model. (B) SEC analysis of SBD641 in the absence (green line) and the presence (orange line) of a 14-fold excess of NR peptide. (C) nESI MS spectrum of SBD641 in the presence of the NR peptide at a protein:peptide ratio of 1:1 at a sample cone voltage of 60 V. Inset is the region of the spectrum showing dimers, at increasing sample cone voltages from front to back. The coloured circles represent charge states that correspond to the same protein species: uncomplexed monomeric protein in dark green, monomeric SBD641 complexed with the NR peptide in light green, the uncomplexed dimeric protein in red, dimeric SBD641 complexed with a single NR peptide molecule in pink, and dimeric SBD641 complexed with two NR peptide molecules in orange. The protein: peptide molar ratio used allowed sufficient resolution of the various charge state series for unambiguous assignment of substrate-bound chaperone states, which are validated by the successive removal of peptide equivalents upon increasing collisional activation (inset). The spectra shown are representative of five repeats, each incorporating >150 scans, with the relative abundances of apo- and holo-forms varying by <5% between individual nanoESI injections. Observed masses differ from those expected, due to residual adduction of solvent molecules and buffer ions, by <1%, as is typical in the spectra of non-covalent complexes (Table S6 in File S1) [57].

To test whether or not the irreversibility of the unfolding of the C-terminal segment of the HLS of the chaperone affects the oligomerization of the protein, we performed analytical SEC experiments on the refolded form of SBD641. The chromatogram obtained (Figure 4C) shows a dramatic increase in the monomeric fraction of the refolded protein compared to the native protein (from 37% to 71%). The difference in the relative fraction of monomeric and oligomeric forms between native and refolded protein is consistent with the degree of irreversibility found for the C-terminal segment of the HLS. Note that the rest of the protein remains structurally and thermodynamically very similar, according to the thermal denaturation behaviour of both conformations of the protein, and that the refolded protein shows the same affinity as the native protein for substrate binding (Figure 4A and Figure 5A). Overall, these results indicate that misfolding of the HLS following thermal denaturation precludes protein oligomerization and suggest that the oligomerization involves the interaction of the interdomain linker with the HLS of the SBD, through a different mechanism from that reported for the binding of a substrate to the SBD binding cleft [10,11] and with helix C playing, probably, an important role, in accord with our thermal unfolding analysis and consistent with previously reported findings [20,32].

### Interaction with substrates

In order to evaluate the influence of oligomerization on substrate binding, we characterized the ability of protein variants with different oligomerization propensities to bind the NR peptide (NRLLLTG) as a model substrate [8,47,48]. The NR peptide was N-terminally labelled with a dansyl fluorophore and complex formation was studied by titrating increasing quantities of the different chaperone variants into solutions containing dansyl-NR peptide and following the fluorescence properties of the dansyl moiety. This procedure has been used previously [10,12,33], but here we show further its suitability for the characterization of substrate binding in Hsp70, despite the presence of the fluorophore at the N-terminal position in the peptide, since essentially the same affinity was found for both dansylated and non-dansylated NR peptide (see Table S4 in File S1 for results and Supplementary Materials and Methods in File S1 for experimental procedure and data analysis of the fluorescence competition assay). The results of the titration experiments using dansylated NR peptide reveal that all the protein variants analyzed are able to bind the NR peptide (Figure 5A).

The full-length SBD (SBD641) was found to bind the NR peptide with an affinity that is closely similar to that of the FL-Hsp70 (*K*_*d*_ = 11 ± 3 µM and 9 ± 3 µM, respectively) and is independent of the presence or absence of the interdomain linker (*K*_*d*_ = 8 ± 2 µM for ΔLSBD641). These results are in agreement with previous studies where the SBD was found to behave independently of the NBD in the nucleotide-free state of both DnaK and human Hsp70 [5,6,49,50]. In contrast, SBD556 was found to have a reduced ability to bind the NR peptide (*K*_*d*_ = 53 ± 16 µM), a feature that has also been found for a similar truncated variant in its bacterial homologue DnaK [12,14]. This result indicates that helices C-E are also important, although not essential, for substrate binding, probably through stabilization of the substrate: chaperone complex as previously proposed [13]. The increase in *K*_*d*_ of a DnaK mutant protein lacking the C-terminal 100 residues (DnaK 2-538, i.e. lacking helices C-E and part of helix B) with respect to wild type DnaK for two different peptide substrates was found to result primarily from a considerable increase in the dissociation rate constants, while the association rate constants were unaffected [3]. All these results indicate that the entire HLS is needed to stabilize the substrate: chaperone complex by preventing complex dissociation. The HLS thus appears to regulate the kinetics of substrate release, which will be further modulated by allosteric communication between the NBD and SBD, depending on the nucleotide status of the NBD, and where the interdomain linker has been found to be of major significance [4,6,51].

### Influence of substrate binding on chaperone oligomerization

It has already been reported that substrate binding to Hsp70 family members results in the disassembly of oligomeric species [15,23,25-27,29]. To investigate whether or not this is also the case in human Hsp70, we performed SEC analysis on different variants of the protein in the absence and the presence of a 14-fold excess of the NR peptide (see results for the SBD641 variant in Figure 5B and for the other variants, including FL-Hsp70, in Figure S10 see Figure S3A, S3C and Supplementary Materials and Methods in File S1 for the validation of the results obtained by SEC using dynamic light scattering (DLS)). In the presence of an excess of the NR peptide, there is a significant increase in the monomeric fraction of the protein in all chaperone variants relative to that in the protein samples in the absence of the peptide. For example, in SBD641, the monomeric fraction increases from 37% to 58% in the presence of peptide (Figure 5B), and consequently, the fractions of dimers and higher-order oligomers are significantly reduced, to 24% and 18% respectively, in comparison with the same fractions in the absence of the peptide (55% and 22%, respectively). It is important to note that, despite the high excess of substrate in comparison with the concentration of chaperone used in these experiments, substrate binding is able to shift the equilibrium towards the accumulation of monomeric chaperone in all protein variants (including FL-Hsp70), but not as completely as would be expected from the values of the affinity constants of the chaperone variants for this substrate, from the relative ratio of substrate: chaperone used here, and from the oligomerization constants extrapolated from the experimental fractions of monomeric and oligomeric species for each protein variant, if the chaperone oligomerization process directly competed for the substrate-binding pocket (i.e. if the substrate-binding pocket were to be part of one of the oligomerization interfaces). For example, for SBD641, ca. 90% of the protein species would be expected to be in the monomeric form (vs the experimentally obtained 58%) according to an equilibrium between monomeric, dimeric and trimeric protein (with an oligomerization dissociation constant ca. 5-10 µM estimated from Table 1) in presence of 14-fold excess of NR peptide (with a dissociation constant of 11 µM, Figure 5A) that would directly compete with chaperone oligomerization for the substrate-binding pocket. Similar findings have also been reported for other Hsp70 homologues [15,26].

To investigate this idea further, we performed nESI MS analysis of SBD641, under native conditions in the presence of the NR peptide, in order to elucidate the stoichiometry of the resulting chaperone/substrate complexes (Figure 5C). Despite the challenges of this type of experiments (see below), peptide binding to both monomeric and dimeric SBD641 is clearly observed, with monomers binding a single peptide, and dimers up to two peptide molecules. Collisional activation of the complexes, achieved by increasing the acceleration voltages in the source region of the mass spectrometer [52], leads to a reduction in the abundance of all the holo forms to a similar extent (i.e. they have disappeared by 200V, see Figure 5C inset), clearly demonstrating the non-covalent binding of the NR peptide. If both the NR peptide and the interdomain linker were to compete for the same interface on the chaperone molecule it would not be possible for a protein dimer to bind two NR peptide molecules. However, with the MS data clearly demonstrating the existence of (SBD641)_2_(NR)_2_ molecules, this is clearly not the case (see also Table S5 and Table S6 in File S1).

We also noted that the interactions responsible for the formation of NR peptide: chaperone complexes are rather labile in the gas phase, resulting in a significant dissociation of the complexes in the MS experiments at all values of the ratio of substrate: chaperone that were used here, consistent with a labile hydrophobic interaction between Hsp70 and the NR peptide (note that this effect was not observed in the MS experiments on the charaterization of the oligomerization level of the different chaperone variants, suggesting that the specific interactions between the NR peptide and the SBD of the chaperone might be intrinsically different from those that take place between the interdomain linker and the SBD). This effect explains the unexpectedly high fractions of unbound chaperone species in the MS experiments. Nonetheless, by using gentle interface conditions we were able to detect different bound species, which has provided valuable qualitative information about the type and stoichiometry of the chaperone species formed in the presence of substrate. Dimeric chaperone species with two NR peptide molecules bound, one in each protein molecule, were observed in the MS experiments, providing direct evidence for the ability of all the protein molecules in the oligomeric chaperone species to bind substrate and, therefore, indicating that the interdomain linker is interacting with a region of the SBD that is not directly involved in substrate binding. All our data suggest that this region is the HLS of the SBD.

An interesting observation in this context is that a crystal structure of an HLS-truncated variant of DnaK (residues 1-504) from *Geobacillus* *kaustophilus* bound to ADP was found to have the linker connecting the NBD and the β–subdomain of the SBD located in the substrate-binding pocket of a neighbouring crystallographic symmetry-related molecule [53]. Although we are not able to certainly exclude that this interaction can occur, we have shown here that it is highly probable that in solution, at least in human Hsp70, the HLS of the SBD plays a fundamental role in promoting the oligomerization of the protein, likely establishing an interaction with the interdomain linker. It is possible that in the particular crystallographic lattice that was examined, the interdomain linker of one molecule happens to be in close proximity to the substrate-binding pocket of a neighbouring molecule, which in turn has a highly exposed hydrophobic substrate-binding pocket as a result of the absence of the HLS. In both the nucleotide-free and ADP-bound states, the full-length protein has been shown to possess a highly flexible linker that is likely to result in a variety of global conformations of the protein; the crystalline state of this truncated protein variant might therefore represent one of the alternative conformations of the protein when the HLS is absent. Indeed, two other crystal structures of Hsp70 homologues containing the NBD, the interdomain linker and the β-sheet subdomain of the SBD but without the HLS, in the presence of ADP (but in the absence of substrate), have been reported and are consistent with this latter conclusion. In the first crystal structure, the SBD interacts directly with the NBD (but not with the interdomain linker) [45], a finding that differs from the results of most studies of ADP-bound Hsp70 homologues [6,50,51]. In the other crystal structure, only the NBD could be detected in the protein crystals, indicating that the interdomain linker and the β-sheet subdomain of the SBD are highly mobile [53], in agreement with results of the studies of the conformation in solution of the ADP-bound or nucleotide-free full-length protein lacking the HLS [13,50,54]. Finally, it is interesting to note that in the vast majority of constructs used for defining NMR and crystal structures of the full-length protein and of the isolated SBDs of Hsp70 homologues, most of the residues of the HLS have been deleted, probably because such constructs have reduced tendencies to oligomerize at the high protein concentrations required for the structural studies; such a conclusion is in agreement with our results suggesting that the HLS is directly involved in chaperone oligomerization.

Our results suggest that the redistribution of oligomeric chaperone species in the presence of an excess of substrate can be attributed to differences in the overall affinity constants for the substrate in the different chaperone oligomeric species instead of a direct competition of oligomerization and substrate binding for the same protein region (the substrate-binding pocket). The affinity for the NR peptide appears to be significantly higher for the monomeric states relative to the oligomeric states of human Hsp70, i.e., there is a preferential binding of the peptide to the monomeric forms of the protein relative to the oligomeric forms. In this way an excess of substrate leads to an enhanced population of monomeric bound species. In the monomeric protein, the bound NR-peptide complex is known to be stabilized by the HLS, with an apparent dissociation constant of 8-10 µM in human FL-Hsp70. In contrast, in the oligomeric species most of the HLSs of the molecules (one out of two in the dimer and two out of three in the trimer) would be interacting with the interdomain linker of their neighbouring molecules and therefore are unable to stabilize the substrate: chaperone complex, which results in a decreased overall affinity for the NR peptide (the *K*_*d*_ obtained for the binding of the NR peptide from the fluorescence titrations of SBD556, the variant without the C-terminal segment of the HLS, is ca. 50 µM; see Figure 5A). Therefore, in the presence of NR peptide, the oligomeric species bound to one substrate molecule would be stabilized relative to complexes of oligomeric species with a larger number of bound substrate molecules, and the bound monomeric species would be preferentially accumulated relative to any oligomeric chaperone species.

## Discussion

Our analysis of a series of carefully designed variants of human Hsp70 has led us to conclude that the interdomain linker connecting the NBD and the SBD represents one of the two protein interfaces of the chaperone oligomerization. Concretely, we have been able to identify a reversible interaction between the linker sequence of one chaperone molecule and the SBD of another molecule that results in the formation of dimers and higher-order oligomers. This interaction, and therefore chaperone oligomerization, depends on protein concentration and temperature, since the irreversible thermal-induced denaturation of the HLS drastically decreases the oligomerization propensity of the chaperone. Different regions of the SBD have been proposed to be involved in this process [17,24,32]. Our data clearly show that the HLS is fundamental for the oligomerization of human Hsp70 and suggest that a direct interaction between this region and the interdomain linker can occur. This last consideration rests in the following experimental evidences: 1) removal of the C-terminal part of the HLS results in a drastic reduction in protein oligomerization, 2) misfolding of the HLS following thermal denaturation of SBD precludes protein oligomerization while maintaining the structural properties of the substrate-binding pocket as well as the affinities for substrates, 3) addition of 14-fold excess of substrate does not shift the equilibrium to monomeric protein as completely as would be expected if the linker would interact with the substrate-binding pocket of the SBD, 4) very similar affinities for the NR peptide were found for two SBD truncated variants that differ only in the presence or absence of the interdomain linker, suggesting that the linker does not compete for the same chaperone region with the NR peptide (note that in these experiments we used a concentration of protein, and hence of the interdomain linker regions, that is 75 times greater than that of the NR peptide, which would result in a dramatic decrease in the apparent affinity of the NR peptide for SBD641 when compared with ∆LSBD641 if there were a direct competition between the linker and the substrate for the substrate-binding pocket; see Figure S11), 5) dimeric chaperone species with two NR peptide molecules bound, one in each protein molecule, were observed in the MS experiments.

**Figure 6:**
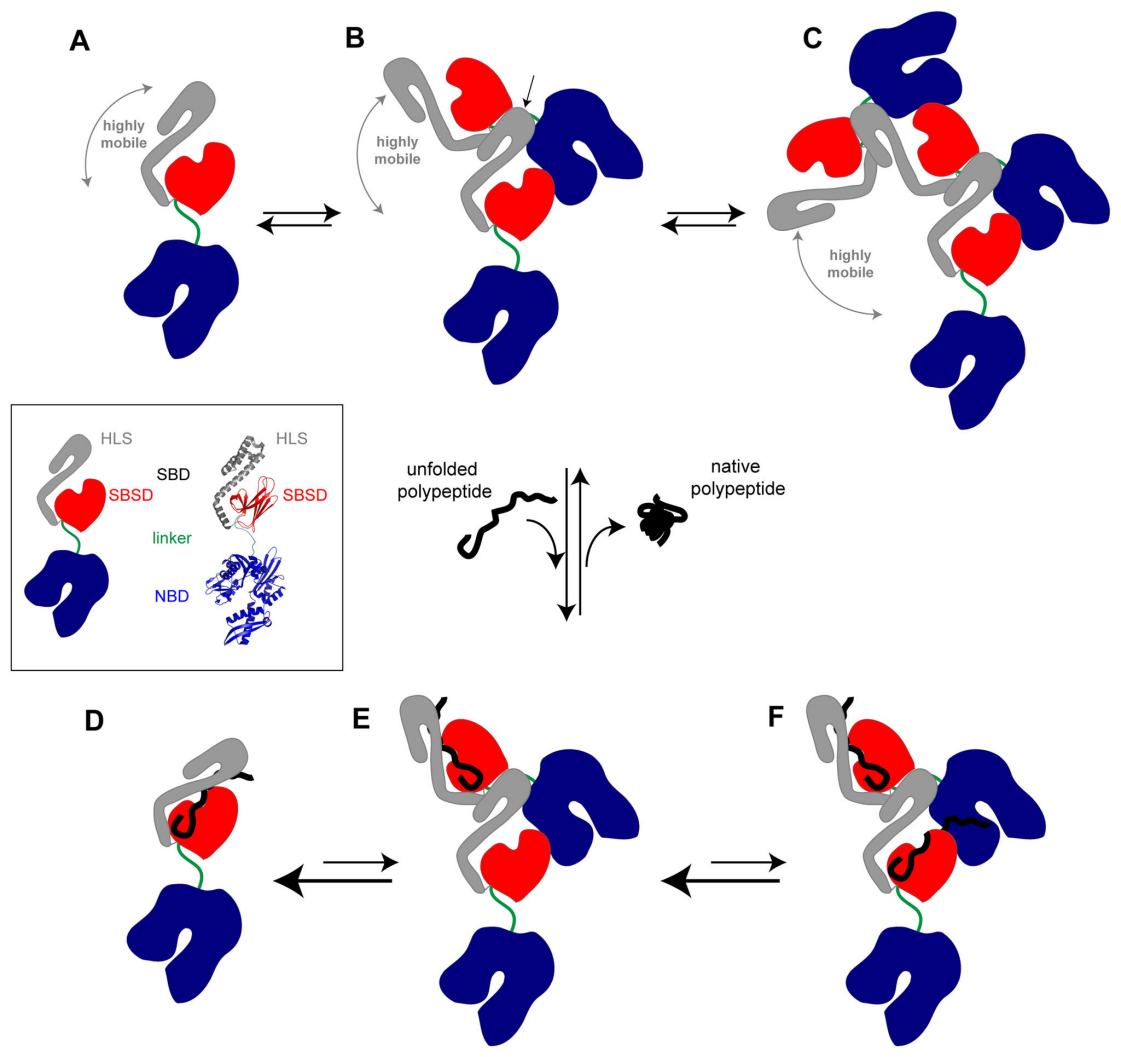
Model for Hsp70 oligomerization. Hsp70 is composed of a nucleotide-binding domain (NBD) and a substrate-binding domain (SBD), which are connected to each other by a highly conserved hydrophobic linker (see insert). The SBD is in turn divided into two subdomains: the substrate-binding subdomain (SBSD), with mostly β-sheet structure, and the helical lid subdomain (HLS) that is rich in α-helical structure. The schematic images of the Hsp70 molecule are based on the crystal structure of DnaK complexed with ADP and substrate (pdb: 2KHO [48]). In the absence of nucleotide and of a polypeptide substrate, Hsp70 exists at equilibrium as a distribution of monomers (state A), dimers (state B), and higher order oligomers (state C). In the monomeric state, the two functional domains are allosterically independent and the HLS of the SBD is highly mobile. Our results show that chaperone oligomerization occurs through an interaction between the HLS of one protein molecule and the interdomain linker of another molecule (this interaction is shown by an arrow in state B), allowing the formation of oligomeric species. In the presence of an unfolded polypeptide substrate, however, the equilibrium between the different states of the chaperone is shifted towards the accumulation of monomeric protein, which is the state that shows the highest affinity for substrates because of the stabilization of the complex by the HLS that acts as a lid preventing substrate release (state D). The oligomers, however, have only one HLS in each species that is free from interactions with the linker of another protein molecule, and are therefore able to stabilize the binding of only one substrate molecule; this protein molecule will, however, be able to stabilize the bound substrate (state E). The remaining HLSs interact with another molecule of the chaperone, and therefore they are unable to act as a lid to their corresponding substrate-binding pockets, resulting in a decreased affinity for substrate (state F). As a consequence of this fact, the overall affinity for the substrate per chaperone molecule is decreased in the oligomeric species when compared with the monomeric protein. Indeed, the higher the order of the oligomer, the higher the number of low-affinity binding pockets per chaperone molecule and the lower the overall affinity of the chaperone molecule for the substrate. Once the substrate folds (to give the native state), its hydrophobic residues are no longer able to interact with the binding pocket of the chaperone, which results in substrate release and the chaperone recovers its initial state (an equilibrium between states A, B and C).

Although we do not yet know the specific interactions that stabilize the oligomeric forms of the chaperone, we note that in human Hsp70 the HLS contains seven leucine residues that are distributed amongst helices B-E. It is therefore possible that these residues could interact with the highly conserved leucine-rich region of the interdomain linker in a manner similar to that observed in other proteins with leucine-rich sequences, such as ribonuclease inhibitors and toll-like receptors [55,56]. If the oligomerization is indeed mediated by leucine/leucine interactions, a significant portion of the HLS and the region around the interdomain linker is likely to be involved in the interaction, as leucine residues span across both the HLS and the interdomain linker and the N-terminal region of the β-sheet subdomain of the SBD. Ideally, crystallographic and/or NMR data on the complete SBD (SBSD + HLS) with the interdomain linker would help to understand the type of interactions responsible for chaperone oligomerization. Unfortunately, no such data is currently available due to the challenges inherent in the crystallization and NMR analysis of this specific domain of the protein.

The relatively large oligomerization interfaces could also explain the fact that various truncated variants of human Hsp70, differing by the number of leucine residues as well as by their accessibility to interact, show different propensities to form oligomers. In the SBD556 truncated variant of human Hsp70, most of the oligomerization interface of the HLS is absent, but the remaining leucine residues of the intact helix B are still able to interact with the interdomain linker. A similar situation takes place in the ΔLSBD641 (and FL-Hsp70 2LD) variant; this variant lacks most of the leucine residues of the oligomerization interface of the interdomain linker, but it is still able to form oligomers, although to a reduced extent, as it still contains two leucine residues close to the interdomain linker with which the HLS can interact. While the NBD, the β-sandwich subdomain in the SBD and the interdomain linker are highly conserved in both sequence and structure across prokaryotes and eukaryotes, the HLS in the SBD is more evolutionary variable both in sequence and structure [40]. This variability in the HLS of the SBD provides an explanation for the differences in oligomerization propensity found for various members of the Hsp70 family despite the extremely high sequence and structure identity [2].

Overall, our results and their interpretation can unify a large number of the findings of previous studies of DnaJ-independent chaperone oligomerization, including the observations (i) that dimers and higher-order oligomers are observed to form both *in vitro* and *in vivo* [15-24,27-31]; (ii) that chaperone oligomerization, involves two different regions of the protein molecules, one of them located in the SBD [17,24,32]; (iii) that Hsp70 homologues display different propensities to oligomerize despite the extremely high sequence and structure similarity within the family [15,16,30]; (iv) that temperature influences chaperone oligomerzation [23,31]; (v) that substrate binding shifts the oligomerization equilibrium towards the accumulation of monomeric protein [15,17]; (vi) that oligomers are able to bind substrates but that the complexes have compromised refolding activities [31]; and (vii) that oligomerization is likely to play a role in the function of the chaperone as a regulatory mechanism [16,23,27-29].

Recent models of the allosteric cycle of Hsp70 propose that the SBD opens and closes periodically in both the ADP-bound and ATP-bound states, and that the differences in substrate association and dissociation rate constants between the two states are a consequence of differing probabilities that the lid opens and forms the substrate-binding site [3]. The mechanism by which the HLS of the SBD changes from the closed to the open conformation to allow substrate binding and dissociation remains unclear, and models involving a small hinge movement [8], or a pivoting of the entire HLS [9] have been proposed.

In the present study, we have focused on the identification of the protein regions involved in Hsp70 oligomerization and on the influence of this process on chaperone substrate binding. We find that Hsp70 oligomers are able to bind substrates with an overall lower affinity than the monomeric form of the protein, where the HLS can act as a lid and stabilize the substrate: chaperone complex. This difference in affinity between protein species results in the accumulation of the functional monomeric protein in the presence of substrates (Figure 6). A recent study by Thompson has shown that DnaK preferentially assembles into small oligomers with limited foldase activity upon ADP binding, and that these complexes then disaggregate into functional monomers upon ADP-ATP exchange [31]. This findings are in agreement with our proposition that chaperone oligomers, which have lower affinities for substrates than the monomer and compromised refolding activities [31], could act as a reservoir of monomers that are ready to function once misfolded substrates are present. In the absence of such substrates, however, the oligomeric species have much reduced activities and could act to prevent both aberrant binding of proteins and unnecessary ATP hydrolysis.

## Supporting Information

File S1 Combined supporting information file of Supplementary Materials and Methods, tables and references. Supplementary Materials and Methods include experimental and data analysis procedures of dynamic light scattering and fluorescence competition assay. Table S1, Comparison of the secondary structure content of the different human Hsp70 variants used in this study derived from far-UV CD spectrum with that expected according to the crystal structure of homologues proteins. Table S2, Assignment of charge state series for nESI MS measurements of protein constructs*a*. Table S3, Thermodynamic parameters of protein thermal denaturation obtained for the different Hsp70 truncated variants. Table S4, Determination of the influence of dansyl fluorophore in the affinity of the NR peptide for Hsp70. Table S5, Assignment of charge state series for nESI MS measurements of SBD641 in the presence of NR peptide. Table S6, Comparison between the theoretical and experimental masses obtained by MSfor SBD641 incubated with NR peptide.
(PDF)

Figure S1 Mass spectra of FL-Hsp70, SBD554, SBD641 and 'C-term' variants under acidified denaturing conditions. The denaturing conditions were achieved using AG 501 –X8 (BioRad) beads (see Materials and Methods section in main text). All protein variants with the exception of the 'C-term' variant, whose spectrum is very similar under native and denaturing conditions, show a mass spectrum that is shifted to the lower *m/z* in comparison to the spectra recorded under aqueous buffered conditions near neutral pH (native conditions), typical for spectra of denatured proteins (see Figure 2 and Materials and Methods section in main text).
(TIF)

Figure S2 Fluorescence titration of the binding of the NR peptide to the different Hsp70 constructs. The absolute difference in fluorescence for all the chaperone variants is shown: SBD556 (red circles), SBD641 (green circles), ΔLSBD641 (black circles), and FL-Hsp70 (blue circles). The continuous lines represent the best fit of the data to a single binding site model. The Fmax value was similar for all the chaperone variants, except for SBD556, which showed ca. a 25% of reduction of the fluorescence intensity. This is likely due to the fact that, as in SBD556 part of the lid is missing, the binding pocket is more exposed to the solvent and therefore the dansyl moiety is less buried in comparison with the other constructs.
(TIF)

Figure S3 Assessment of the populations of oligomeric species in SBD641 obtained by SEC. The influence of the sample incubation time on the distribution of oligomeric species obtained by SEC was first analyzed (A). The samples were incubated for 1 h (black line and red line representing the chaperone variant alone or in presence of 14-times excess of the NR peptide) and for 4 h (grey line and orange line for the absence and presence of the NR peptide, respectively) before their analysis, and the obtained chromatograms were found essentially identical (within experimental error) and then independent of the incubation time within the 1-4 h range, indicating that the equilibrium between oligomeric species is reached before 1 h incubation both in presence and absence of the NR peptide substrate. The relative populations of oligomeric species obtained by SEC were then validated by comparing the fraction of oligomeric species so obtained with the apparent hydrodynamic diameter (D_H_) of the mixture between monomers and oligomers in SBD641 obtained by dynamic light scattering (DLS) as a function of chaperone concentration (B) and ratio between chaperone concentration and NR peptide (C) (see Supplementary Materials and Methods in File S1 for the experimental procedure). The experimental values obtained for the apparent D_H_ by DLS are represented with black squares (mean and standard deviation of 3 measurements), while the fraction of oligomeric species obtained by SEC are depicted by red circles (experimental errors are also shown). DLS is unable to resolve the different SBD641 oligomeric species (monomers, dimers, trimers and tetramers), which are all engulfed in a unique peak in the obtained experimental intensity size distribution. The apparent D_H_ of the protein in these cases does not correspond to any individual protein species nor to a linear combination of the values of the different species weighted for their relative population (the intensity of the signal is proportional to the sixth power of the diameter of the individual species), however, it correlates with the amount of oligomeric species in the sample so that the higher the amount of oligomeric species, the higher the apparent D_H_ obtained by DLS. In this way we can delimitate the protein concentration range at which the D_H_ increases and relate the increase in D_H_ with the increase in relative population of oligomers obtained by SEC as a function of protein concentration. As seen in panel (B), not only the protein concentration range at which D_H_ increases coincides with that at which the fraction of oligomers was found to increase by SEC, but also, both techniques give rise to a very similar dependency of oligomer population with protein concentration, indicating that the values for the fraction of oligomeric species obtained by SEC are good estimates of the actual relative populations of species at the initial protein concentration and that minor oligomer dissociation occurs during the chromatography. Lastly, the same approach was used to evaluate the populations of oligomeric species of SBD641 obtained by SEC in absence and presence of different concentrations of NR peptide (20 µM of SBD641 and 0, 20, 100 and 300 µM of NR peptide were used) (C). The apparent D_H_ obtained by DLS in the presence of increasing concentrations of NR peptide was found to decrease slightly, even at a protein:peptide ratio of 1:15, in agreement with the reduction of oligomer fraction estimated by SEC at this conditions (from ca. 0.7 to 0.5 upon incubation with 14-times excess of NR peptide). The apparent D_H_ obtained by DLS and the fraction of oligomeric species estimated by SEC (ca. 0.1) of ΔLSBD641 at the same protein concentration were also added to the plot. For an excess of 14-times substrate concentration with respect to chaperone concentration, an almost total reduction of oligomers would be expected if the substrate would compete with chaperone oligomerization for the same protein region (direct competition for the same binding site) with similar affinities (both substrate binding and oligomerization in SBD641 show dissociation constants of the order of 1-10 µM; see Figure 5A for substrate binding and the extrapolation to a 0.5 oligomer fraction in panel B), which is not in agreement with the experimental apparent D_H_ and oligomer fraction obtained by DLS and SEC, respectively.
(TIF)

Figure S4 Concentration dependency of the analytical SEC chromatograms of the 'C-term' variant. The protein concentrations used were 1µM (black line), 2µM (orange line), 5 µM (pink line), 10 µM (cyan line), 70 µM (red line) and 125 µM (green line). Essentially the same elution profile was found for all the protein concentrations used. At the top the chromatogram the previously reported Stokes radii of the standard proteins used to calibrate the column are reported: conalbumin (40.4 Å), ovalbumin (30.5 Å), carbonic anhydrase (23.6 Å) [S3], ribonuclease A (16.4 Å).
(TIF)

Figure S5 Analytical SEC analysis of the different protein constructs at 70 µM of protein concentration. FL-Hsp70 (A), SBD641 (B), SBD556 (C) 'C-term' (D) and ∆LSBD641 (E).
(TIF)

Figure S6 Analytical SEC analysis of the different oligomeric fractions of FL-Hsp70 (A), SBD556 (B), SBD641 (C) and ∆LSBD641 (D). The monomeric and oligomeric peaks isolated by SEC (upper panel) were re-loaded into the same column for analysis (middle and bottom panels, respectively).
(TIF)

Figure S7 Mass spectra of ΔLSBD641 under acidified denaturing (**upper panel**) and native conditions (**bottom panel**). This protein variant behaves similarly to SBD641 under denaturing conditions, while it shows a reduced propensity to oligomerize under native conditions.
(TIF)

Figure S8 (A) Far-UV CD thermally-induced denaturation of the native and refolded protein ∆LSBD641 (black and grey). (B) Tryptophan (W580) fluorescence spectra of ∆LSBD641 in its native (black), thermally denatured (black dashed line) and refolded states (grey continuous lines); the spectrum of SBD556 in its native state is shown as a negative control (red continuous line). (C) Thermal denaturation of the 'C-term' variant followed by CD at 222nm (orange points). Violet points show the thermal denaturation curve of the refolded protein. Both experimental curves were fitted to a two-state model; the best fit is shown as continuous line. (D) Tryptophan fluorescence emission spectra of the 'C-term' variant in its native (Orange continuous line), thermally denatured (black dashed line) and refolded conformations (violet continuous line). The spectrum of SBD556 is shown in red continuous line as a negative control.
(TIF)

Figure S9 **Comparison of the far-UV CD spectrum of FL-Hsp70 (in blue) and FL-Hsp70 2LD (in cyan) at 7 µM of protein concentration.**
(TIF)

Figure S10 Analytical SEC each for FL-Hsp70 (A), SBD556 (B) and ΔLSBD641 (C) in the presence of a 14-fold excess of the NR peptide (**orange continuous lines**) in comparison with the profiles obtained for the free protein. At the top of each chromatogram the molecular weights of the standard proteins used to calibrate the column are reported.
(TIF)

Figure S11 Comparison between the experimental titration curve of SBD641 (**green circles and line**) and the theoretical titration curves expected if the interdomain linker would compete with the NR peptide for the substrate-binding pocket of the chaperone assuming a range of affinity constants for the interaction with the linker (**grey area**). The experimental titration curve of ΔLSBD641 is also shown as reference (black circles and line). The theoretical curves were simulated assuming a competitive model (competition between the linker and the NR peptide for the same chaperone region), solving the law of mass action equation for the equilibrium 2E +S + I (-) ES + EI, where E represents SBD641, S represents the NR peptide and I represents the linker. The total concentrations of species used in the simulation were the same as those used in the experimental titration: [S] = 2µM, [E] = [I] = 0-150 μM. The dissociation constant for the interaction of the NR peptide with the chaperone (K_d_(ES) = 8 µM) was obtained from the experimental titration of ΔLSBD641 (variant without linker), and the dissociation constant for the interaction of the linker with SBD in SBD641 was estimated from Table 1 (K_d_(EI) = 5-10 µM).
(TIF)
