# Supplementary material for: Hsp70 Oligomerization Is Mediated by an Interaction between the Interdomain Linker and the Substrate-Binding Domain
Source: PLoS One. 2013 Jun 28;8(6):e67961. doi: 10.1371/journal.pone.0067961 (PMC3696110; doi:10.1371/journal.pone.0067961)
Supplement: File S1 — Supplementary Materials and Methods include experimental and data analysis procedures of dynamic light scattering and fluorescence competition assay. Table S1, Comparison of the secondary structure content of the different human Hsp70 variants used in this study derived from far-UV CD spectrum with that expected according to the crystal structure of homologues proteins. Table S2, Assignment of charge state series for nESI MS measurements of protein constructsa. Table S3, Thermodynamic parameters of protein thermal denaturation obtained for the different Hsp70 truncated variants. Table S4, Determination of the influence of dansyl fluorophore in the affinity of the NR peptide for Hsp70. Table S5, Assignment of charge state series for nESI MS measurements of SBD641 in the presence of NR peptide. Table S6, Comparison between the theoretical and experimental masses obtained by MSfor SBD641 incubated with NR peptide. (PDF) [file pone.0067961.s001.pdf]

Hsp70 Oligomerization is Mediated by an Interaction between the Interdomain Linker and the Substrate-Binding Domain

Francesco A. Aprile, Anne Dhulesia, Florian Stengel, Cintia Roodveldt, Justin L Benesch, Paolo Tortora, Carol V. Robinson, Xavier Salvatella, Christopher M. Dobson and Nunilo Cremades

Correspondence should be addressed to N.C. (nc347@cam.ac.uk).

Supporting Information

Supplementary Materials and Methods

Dynamic light scattering. Representative size distributions of SBD641 at different protein concentrations in Tris 50 mM, pH 7.4, 150 mM KCl, 5 mM $MgCl_2$ were recorded at 25 ºC on the Zetasizer Nano ZS instrument (Malvern Instruments Ltd.) at 633 nm after 1 h of incubation at room temperature. Alterations on the size distribution by substrate binding were also tested at exactly the same conditions, incubating 16 μM of SBD641 in the presence of different concentrations of the NR peptide (ratio 1:1, 1:5 and 1:15). Some samples were analyzed after 1 h and 4 h incubation at room temperature and the same intensity size distributions were obtained. The samples were centrifuged for 15 min at 13200 rpm to remove dust particles and the scattered light was detected at an angle of 173°. The acquired data were analyzed by Zetasizer Nano software (Malvern Instruments Ltd.).

Fluorescence competition assay. Samples containing 50 μM ΔLSBD641 and 2 μM dansylated-NR peptide (D-NR) in Tris 50 mM, pH 7.4, 150 mM KCl, 5 mM $MgCl_2$ were incubated in the presence of different concentrations of non-dansylated NR peptide (ND-NR; 0, 50 and 200 μM) for 1h at room temperature and the dansyl fluorescence emission spectrum of each sample was recorded from 400 to 630 nm after excitation at 330 nm. The intensity at the maximum emission wavelength of three different samples for which ten spectra were recorded was averaged, and the fraction of bound D-NR peptide was then determined experimentally from the reduction in the fluorescence signal in relation to the reference sample without ND-NR peptide for which the fraction of bound D-NR peptide is known (83% of the D-NR peptide is bound in this conditions according to the affinity measured by fluorescence titration; see Figure 5a in main text).

The fraction of bound D-NR peptide was theoretically calculated for each sample according to a competitive model where the two ligands bind to the same substrate-binding pocket in the chaperone and assuming different values for the affinity of the ND-NR peptide for ΔLSBD641 (K_d = 4, 8, 12, 16, 24 μM; the K_d for D-NR peptide obtained experimentally by direct fluorescence titration was 8 ± 2 μM).

From the mass balance of the total protein concentration (P_T) in a two-ligands competitive model and substituting the concentration of each complex (PL_1 and PL_2, corresponding to the complex with D-NR and ND-NR peptide, respectively) by the expression obtained from the definition of the different dissociation constants, a third-order polynomial expression ($A \cdot P^3 + B \cdot P^2 + C \cdot P + D = 0$) of the free concentration of protein (P) is obtained, where the coefficients of the different terms are:

$$A = 1; B = K_{d1} + K_{d2} + L_{1T} + L_{2T} - P_T; C = K_{d1}K_{d2} + L_{1T}K_{d2} + L_{2T}K_{d1} - k_{d1}P_T - K_{d2}P_T;$$
$$D = -K_{d1}K_{d2}P_T$$

where K_{d1} = 8 μM, the dissociation constant of the D-NR peptide:ΔLSBD641 complex, K_{d2} is the dissociation constant of the ND-NR peptide:ΔLSBD641 complex, P_T = 50 μM, the total concentration of ΔLSBD641, L_{1T} = 2 μM, the total concentration of D-NR peptide , and L_{2T} is the total concentration of ND-NR peptide.

Supplementary Tables

Table S1. Comparison of the secondary structure content of the different human Hsp70 variants used in this study derived from far-UV CD spectrum with that expected according to the crystal structure of homologues proteins.

	Predicted from crystal structure[a] (%)			Estimated from far-UV CD[b] (%)		
	α-helix	β-sheet	Random-coil	α-helix	β-sheet	Random-coil
**SBD556**	22.6	26.9	50.5	21	25	54
**SBD641**	37.4	21.8	40.8	33	18	49
**ΔLSBD641**	37.4	22.5	40.1	35	18	47
**C-term**	64	0	36	60	7	33
**FL-Hsp70**	39.8	22.1	38.1	45	23	32

[a] NBD (PDB ID: **1HJO** [S1]) and SBD (PDB ID: **1DKX** [8])

[b] K2D software was used for spectra deconvolution [35,S2].

Table S2. Assignment of charge state series for nESI MS measurements of protein constructs[a].

FL Hsp 70	Monomeric			Dimeric			3 mer			4mer			Sum	Percentage			
Fig. 2C	m/z	m/z	m/z	m/z	m/z	m/z	m/z	m/z	m/z	m/z	m/z	m/z		Momomer	Dimer	3 mer	4 mer
Peak	3937	4169	4433	5456	5675	5912											
Charge State	18+	17+	16+	23+	22+	21+											
Rel Intensity	325	280	55	5	6	3							674	98	2	0	0
**SBD 641**	**Monomeric**			**Dimeric**			**3 mer**			**4mer**			**Sum**	**Percentage**			
Fig. 2D	m/z	m/z	m/z	m/z	m/z		m/z	m/z	m/z	m/z	m/z	m/z		Momomer	Dimer	3 mer	4 mer
Peak	2596	2851	3151	3550	3787	4058	4253	4485	4733	5159	5412	5677					
Charge State	11+	10+	09+	16+	15+	14+	20+	19+	18+	22+	21+	20+					
Rel Intensity	32	52	12	33	36	13	6	12	7	2	1	1	207	46	40	12	2
**ΔLSBD641**	**Monomeric**			**Dimeric**			**3 mer**			**4mer**			**Sum**	**Percentage**			
Fig. 3C	m/z	m/z	m/z	m/z	m/z	m/z	m/z	m/z	m/z	m/z	m/z	m/z		Momomer	Dimer	3 mer	4 mer
Peak	2476	2724		3638	3903												
Charge State	11+	10+		15+	14+												
Rel Intensity	216	243		22	16								497	92	8		
**SBD556**	**Monomeric**			**Dimeric**			**3 mer**			**4mer**			**Sum**	**Percentage**			
Fig. 2G	m/z	m/z	m/z	m/z	m/z	m/z	m/z	m/z	m/z	m/z	m/z	m/z		Momomer	Dimer	3 mer	4 mer
Peak	2174	2446	2794	3002	3251	3547	3904	4181									
Charge State	9+	8+	7+	13+	12+	11+											
Rel Intensity	114	240	17	9	41	21	7	5					454	82	16	3	0
**C-term**	**Monomeric**			**Dimeric**			**3 mer**			**4mer**			**Sum**	**Percentage**			
Fig. 2H	m/z	m/z	m/z	m/z	m/z	m/z	m/z	m/z	m/z	m/z	m/z	m/z		Momomer	Dimer	3 mer	4 mer
Peak	1396	1595		2526													
Charge State	7+	6+		9+													
Rel Intensity	28	43											71	100			

[a] Nanoflow electrospray mass spectra of the different protein constructs: FL-Hsp70, SBD641, ΔLSBD641, SBD556 and C-term used in this study. Shown are the charge states used for unambiguous assignment and quantification. Color coding as in the main text is used: charge states series assigned to the monomers are shown in green. Dimers, 3mers and 4mers are shown in red, blue and yellow, respectively

Table S3. Thermodynamic parameters[a] of protein thermal denaturation obtained for the different Hsp70 truncated variants.

	1st transition		2nd transition	
	ΔH_m	T_m	ΔH_m	T_m
**SBD556**	63.9 ± 1.9	46.8 ± 0.1	85.4 ± 2.6	64.5 ± 0.1
**SBD641**	49.4 ± 3.3	45.6 ± 0.4	53.3 ± 1.2	73.1 ± 0.1
**ΔLSBD641**	45.6 ± 2.4	44.9 ± 0.3	61.0 ± 1.1	72.3 ± 0.1
**C-term**	51.5 ± 1.5	49.9 ± 0.2	-	-

[a] ΔH_m is given in $kcal.mol^{-1}$ and T_m in °C.
The errors reported in the table represent the standard errors of the fits.

Table S4. Determination of the influence of dansyl fluorophore in the affinity of the NR peptide for Hsp70 [a].

Ratio ΔLSBD641:ND-NR	Fraction of bound dansylated NR peptide					
	Experimentally determined [b]	Theoretically estimated [c] $K_{d\_ND\text{-}NR}$ (μM)				
		4	8	12	16	24
**1:0**	0.83	-	-	-	-	-
**1:1**	0.62 ± 0.1	0.59	0.66	0.7	0.72	0.75
**1:4**	0.35 ± 0.1	0.14	0.24	0.31	0.37	0.45

[a] The influence of the dansyl moiety on the affinity of the NR peptide was determined by a competitive assay between dansylayed and non-dansylated NR peptide (D-NR, ND-NR, respectively).
[b] See Supp. Materials and Methods.
[c] The theoretical fraction of D-NR peptide bound in presence of ND-NR peptide was determined according to a competitive model where the two ligands bind to the same protein binding site and assuming different affinities for the binding of the ND-NR peptide for the chaperone. The good agreement between the experimental and the theoretical values assuming an affinity for the ND-NR peptide similar to the affinity found for the D-NR peptide (K_d ca. 8 μM) demonstrates that dansylated and non-dansylated NR peptide have very similar (if not the same) affinities and further proves the use of dansyl fluorophore to assay the binding of substrates in Hsp70.

Table S5. Assignment of charge state series for nESI MS measurements of SBD641 in the presence of NR peptide[a].

SBD641+ NR petide																	
**SBD641**	**Monomeric**			**Dimeric**			**3 mer**			**4mer**			**Sum**	**Percentage**			
**Fig. 7**	m/z	m/z	m/z	m/z	m/z	m/z	m/z	m/z	m/z	m/z	m/z	m/z		Momomer	Dimer	3 mer	4 mer
Peak	2573	2830	3144	3535	3770	4039	4248	4472		4350	4526	4712					
Charge State	11+	10+	9+	16+	15+	14+	20+	19+		26+	25+						
Rel Intensity	92	179	99	69	65	34	12	15		9	12		586	63	29	5	4
**SBD 641 + NR Peptide**	**Monomeric + NR peptide**			**Dimeric + NR peptide**			**3 mer + NR peptide**			**4mer + NR peptide**			**Sum**	**Percentage**			
**Fig. 7**	m/z	m/z	m/z	m/z	m/z		m/z	m/z	m/z	m/z	m/z	m/z		Momomer	Dimer	3 mer	4 mer
Peak	2665	2933	3257	3598	3838	4113											
Charge State	11+	10+	9+	16+	15+	14+											
Rel Intensity	29	16	5	49	33	9							141	35	65	0	0
**SBD 641 + 2 NR Peptide**	**Monomeric + 2 NR peptide**			**Dimeric + 2 NR peptide**			**3 mer + 2 NR peptides**			**4mer + 2 NR peptides**			**Sum**	**Percentage**			
**Fig. 7**	m/z	m/z	m/z	m/z	m/z		m/z	m/z	m/z	m/z	m/z	m/z		Momomer	Dimer	3 mer	4 mer
Peak				3665													
Charge State				16+	15+	14+											
Rel Intensity				6	3								9	0	100	0	0

[a] Nanoflow electrospray mass spectra of SBD641in presence of NR peptide in a ratio 1:1 . Shown are the charge states used for unambiguous assignment and quantification. Color coding as in the main text is used.

Table S6. Comparison between the theoretical and experimental masses obtained by MS for SBD641 incubated with NR peptide.

	Mass (calc) in Da	Mass (measured) in Da	Error (calc vs measured) in %
Monomeric	28209	28307	0.346
Dimeric	56418	56536	0.209
3 mer	84627	84942	0.371
4mer	112836	113091	0.225
NR peptide	1020		
Monomeric + NR peptide	29229	29323	0.321
Monomeric + 2 NR peptide	30249	not identified in spectum	
Dimeric + NR peptide	57438	57552	0.198
Dimeric + 2 NR peptide	58458	58548	0.154
3 mer + NR peptide	85647	85973	0.379
3 mer + 2 NR peptides	86667	not identified in spectum	
4mer + NR peptide	113856	not identified in spectum	
4mer + 2 NR peptides	114876	not identified in spectum	

Supplementary References

1. Osipiuk J, Walsh MA, Freeman BC, Morimoto RI, Joachimiak A (1999) Structure of a new crystal form of human Hsp70 ATPase domain. Acta Crystallogr D Biol Crystallogr 55: 1105-1107.

2. Merelo JJ, Andrade MA, Prieto A, Morán F (1994) Proteinotopic feature maps. Neurocomputing 6: 443-454.

3. Toyota H, Hosokawa M, Urabe I, Yomo T (2008) Emergence of polyproline II-like structure at early stages of experimental evolution from random polypeptides. Mol Biol Evol 25: 1113-1119.
