# Supplementary figures and images for: Hsp70 Oligomerization Is Mediated by an Interaction between the Interdomain Linker and the Substrate-Binding Domain
Source: PLoS One. 2013 Jun 28;8(6):e67961. doi: 10.1371/journal.pone.0067961 (PMC3696110; doi:10.1371/journal.pone.0067961)

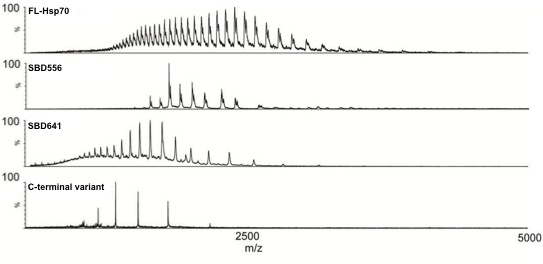

Supplement: Figure S1 — The denaturing conditions were achieved using AG 501 –X8 (BioRad) beads (see Materials and Methods section in main text). All protein variants with the exception of the 'C-term' variant, whose spectrum is very similar under native and denaturing conditions, show a mass spectrum that is shifted to the lower m/z in comparison to the spectra recorded under aqueous buffered conditions near neutral pH (native conditions), typical for spectra of denatured proteins (see Figure 2 and Materials and Methods section in main text). (TIF) [file pone.0067961.s002.tif]

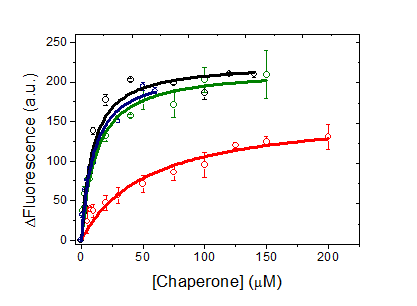

Supplement: Figure S2 — The absolute difference in fluorescence for all the chaperone variants is shown: SBD556 (red circles), SBD641 (green circles), ΔLSBD641 (black circles), and FL-Hsp70 (blue circles). The continuous lines represent the best fit of the data to a single binding site model. The Fmax value was similar for all the chaperone variants, except for SBD556, which showed ca. a 25% of reduction of the fluorescence intensity. This is likely due to the fact that, as in SBD556 part of the lid is missing, the binding pocket is more exposed to the solvent and therefore the dansyl moiety is less buried in comparison with the other constructs. (TIF) [file pone.0067961.s003.tif]

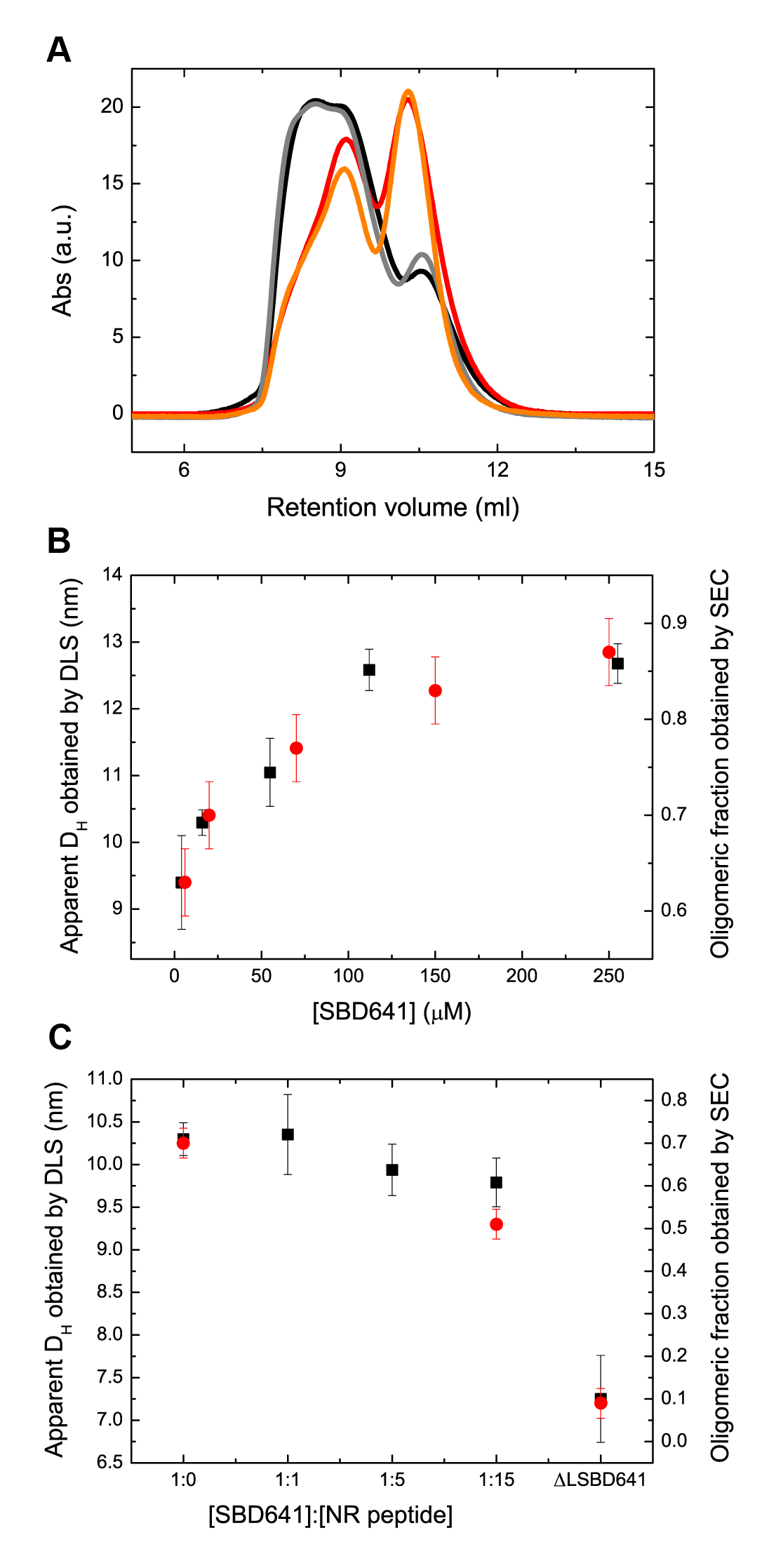

Supplement: Figure S3 — The influence of the sample incubation time on the distribution of oligomeric species obtained by SEC was first analyzed (A). The samples were incubated for 1 h (black line and red line representing the chaperone variant alone or in presence of 14-times excess of the NR peptide) and for 4 h (grey line and orange line for the absence and presence of the NR peptide, respectively) before their analysis, and the obtained chromatograms were found essentially identical (within experimental error) and then independent of the incubation time within the 1-4 h range, indicating that the equilibrium between oligomeric species is reached before 1 h incubation both in presence and absence of the NR peptide substrate. The relative populations of oligomeric species obtained by SEC were then validated by comparing the fraction of oligomeric species so obtained with the apparent hydrodynamic diameter (DH) of the mixture between monomers and oligomers in SBD641 obtained by dynamic light scattering (DLS) as a function of chaperone concentration (B) and ratio between chaperone concentration and NR peptide (C) (see Supplementary Materials and Methods in File S1 for the experimental procedure). The experimental values obtained for the apparent DH by DLS are represented with black squares (mean and standard deviation of 3 measurements), while the fraction of oligomeric species obtained by SEC are depicted by red circles (experimental errors are also shown). DLS is unable to resolve the different SBD641 oligomeric species (monomers, dimers, trimers and tetramers), which are all engulfed in a unique peak in the obtained experimental intensity size distribution. The apparent DH of the protein in these cases does not correspond to any individual protein species nor to a linear combination of the values of the different species weighted for their relative population (the intensity of the signal is proportional to the sixth power of the diameter of the individual species), however, it correlate [file pone.0067961.s004.tif]

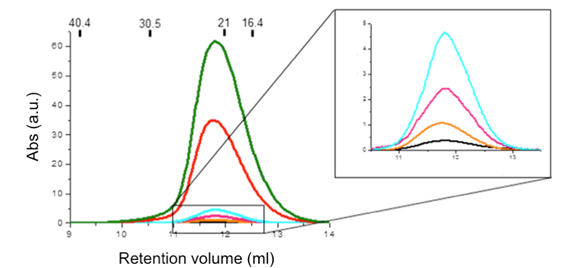

Supplement: Figure S4 — The protein concentrations used were 1µM (black line), 2µM (orange line), 5 µM (pink line), 10 µM (cyan line), 70 µM (red line) and 125 µM (green line). Essentially the same elution profile was found for all the protein concentrations used. At the top the chromatogram the previously reported Stokes radii of the standard proteins used to calibrate the column are reported: conalbumin (40.4 Å), ovalbumin (30.5 Å), carbonic anhydrase (23.6 Å) [S3], ribonuclease A (16.4 Å). (TIF) [file pone.0067961.s005.tif]

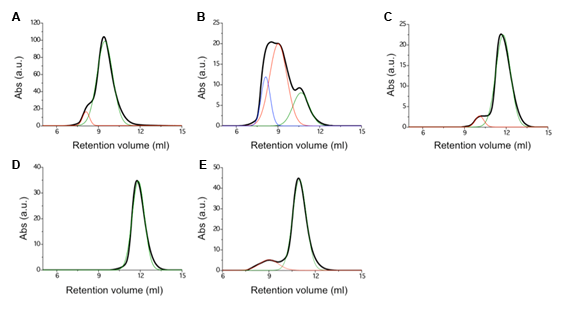

Supplement: Figure S5 — FL-Hsp70 (A), SBD641 (B), SBD556 (C) 'C-term' (D) and ∆LSBD641 (E). (TIF) [file pone.0067961.s006.tif]

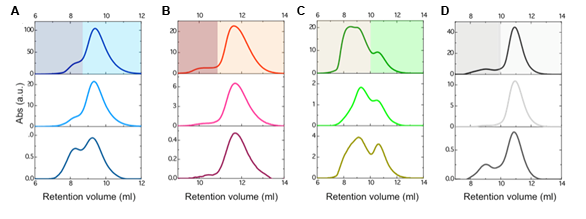

Supplement: Figure S6 — The monomeric and oligomeric peaks isolated by SEC (upper panel) were re-loaded into the same column for analysis (middle and bottom panels, respectively). (TIF) [file pone.0067961.s007.tif]

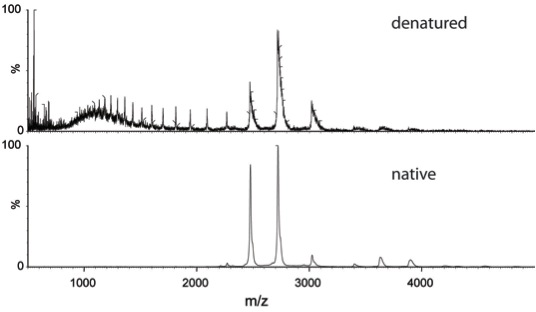

Supplement: Figure S7 — This protein variant behaves similarly to SBD641 under denaturing conditions, while it shows a reduced propensity to oligomerize under native conditions. (TIF) [file pone.0067961.s008.tif]

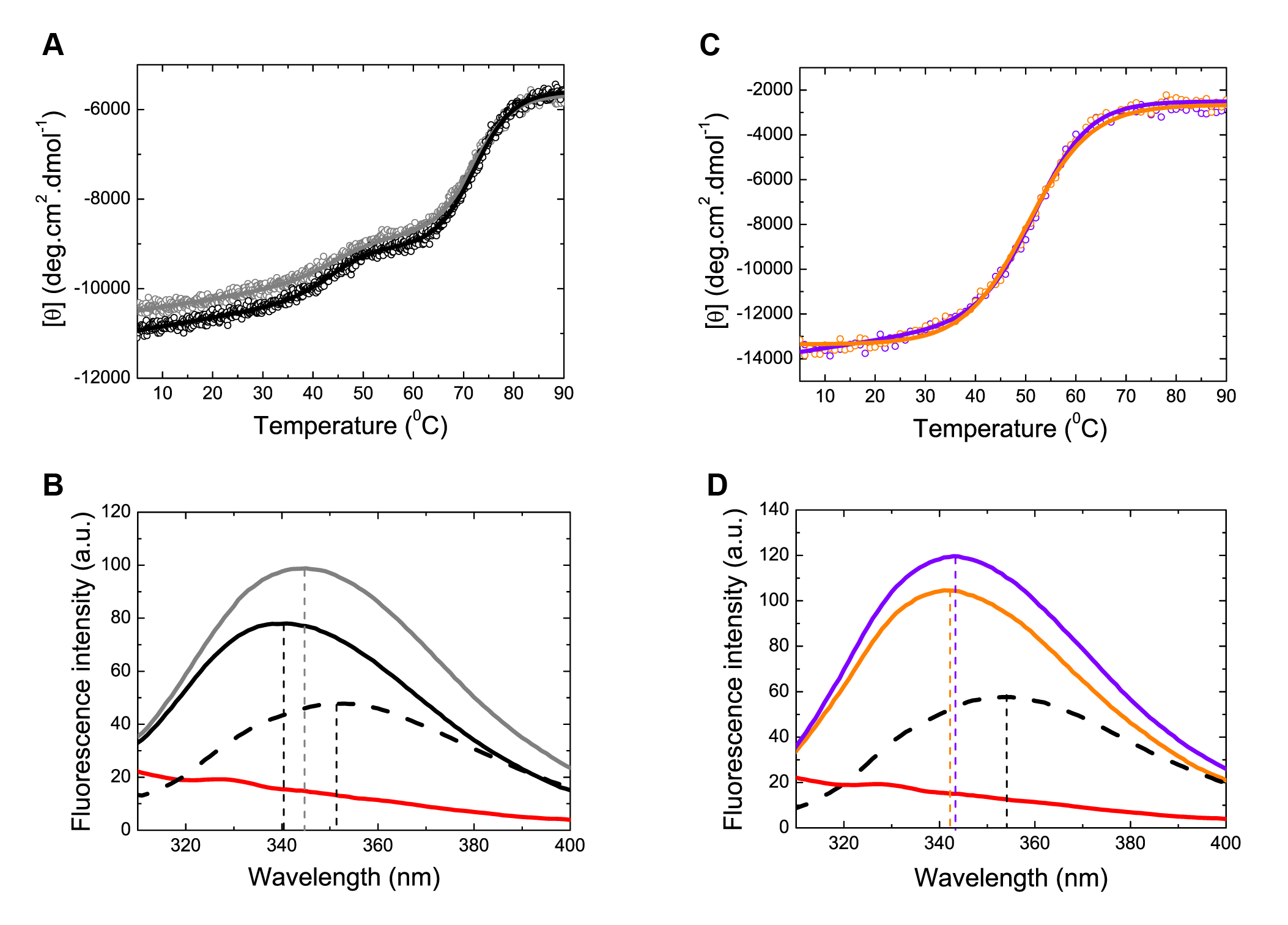

Supplement: Figure S8 — (A) Far-UV CD thermally-induced denaturation of the native and refolded protein ∆LSBD641 (black and grey). (B) Tryptophan (W580) fluorescence spectra of ∆LSBD641 in its native (black), thermally denatured (black dashed line) and refolded states (grey continuous lines); the spectrum of SBD556 in its native state is shown as a negative control (red continuous line). (C) Thermal denaturation of the 'C-term' variant followed by CD at 222nm (orange points). Violet points show the thermal denaturation curve of the refolded protein. Both experimental curves were fitted to a two-state model; the best fit is shown as continuous line. (D) Tryptophan fluorescence emission spectra of the 'C-term' variant in its native (Orange continuous line), thermally denatured (black dashed line) and refolded conformations (violet continuous line). The spectrum of SBD556 is shown in red continuous line as a negative control. (TIF) [file pone.0067961.s009.tif]

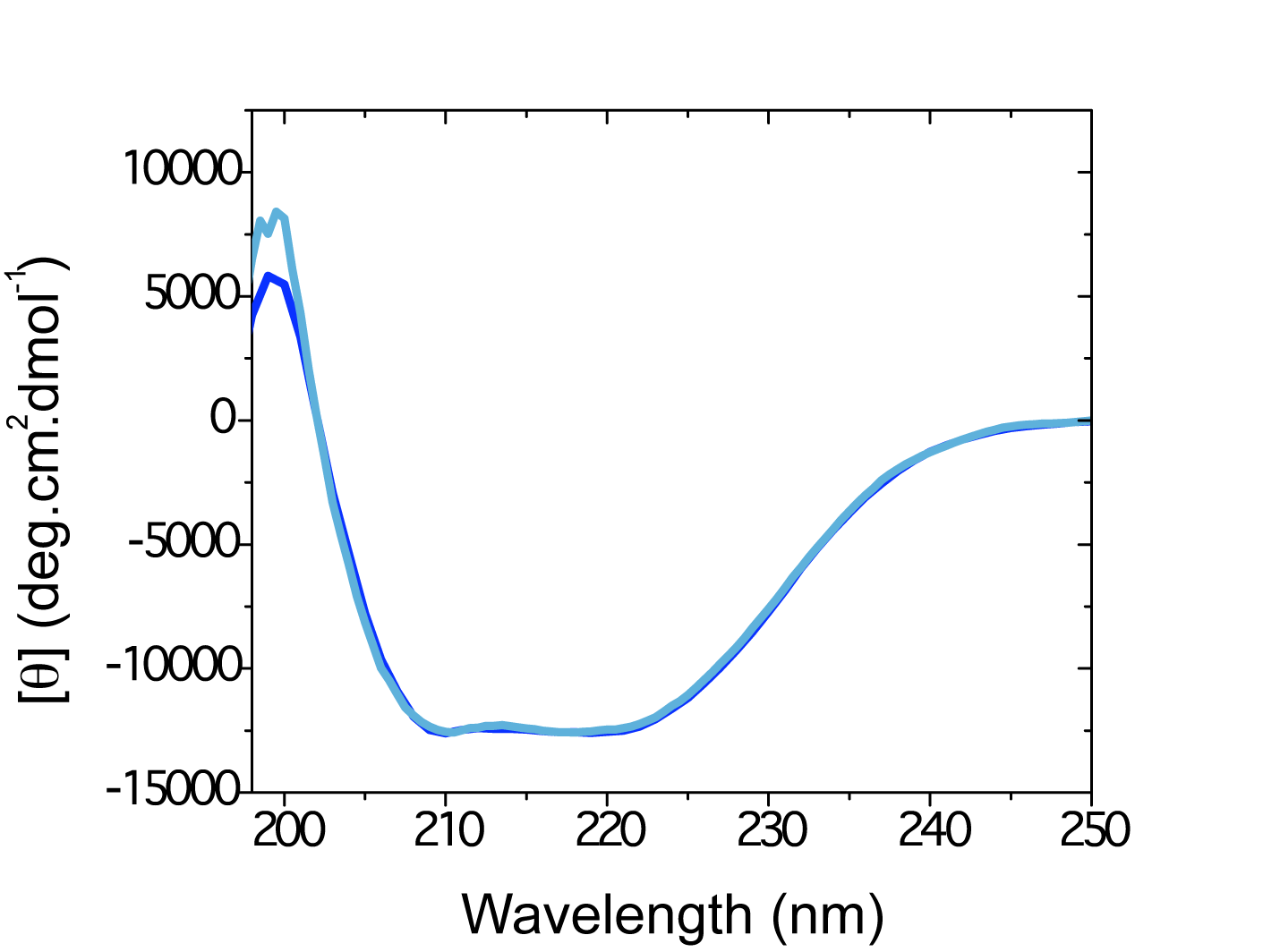

Supplement: Figure S9 — Comparison of the far-UV CD spectrum of FL-Hsp70 (in blue) and FL-Hsp70 2LD (in cyan) at 7 µM of protein concentration. (TIF) [file pone.0067961.s010.tif]

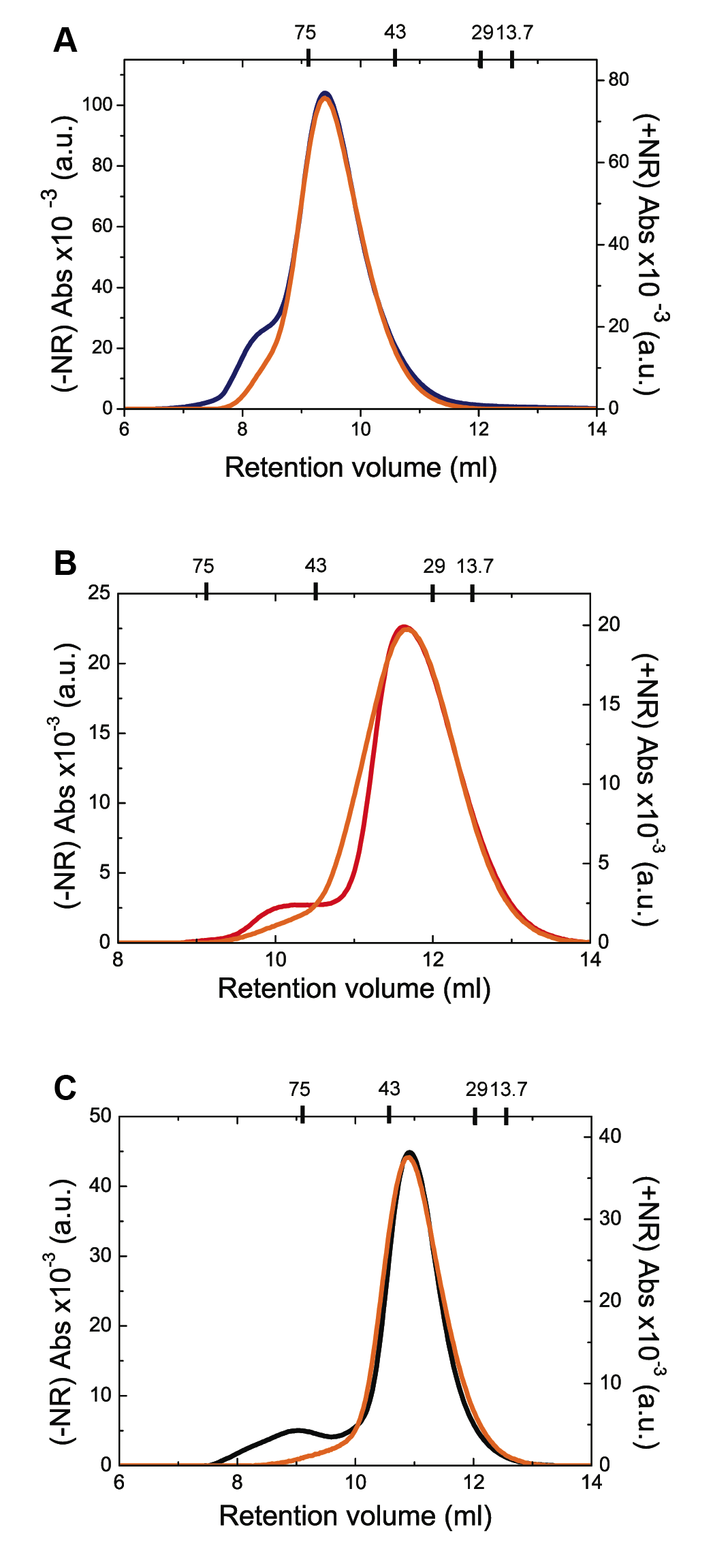

Supplement: Figure S10 — At the top of each chromatogram the molecular weights of the standard proteins used to calibrate the column are reported. (TIF) [file pone.0067961.s011.tif]

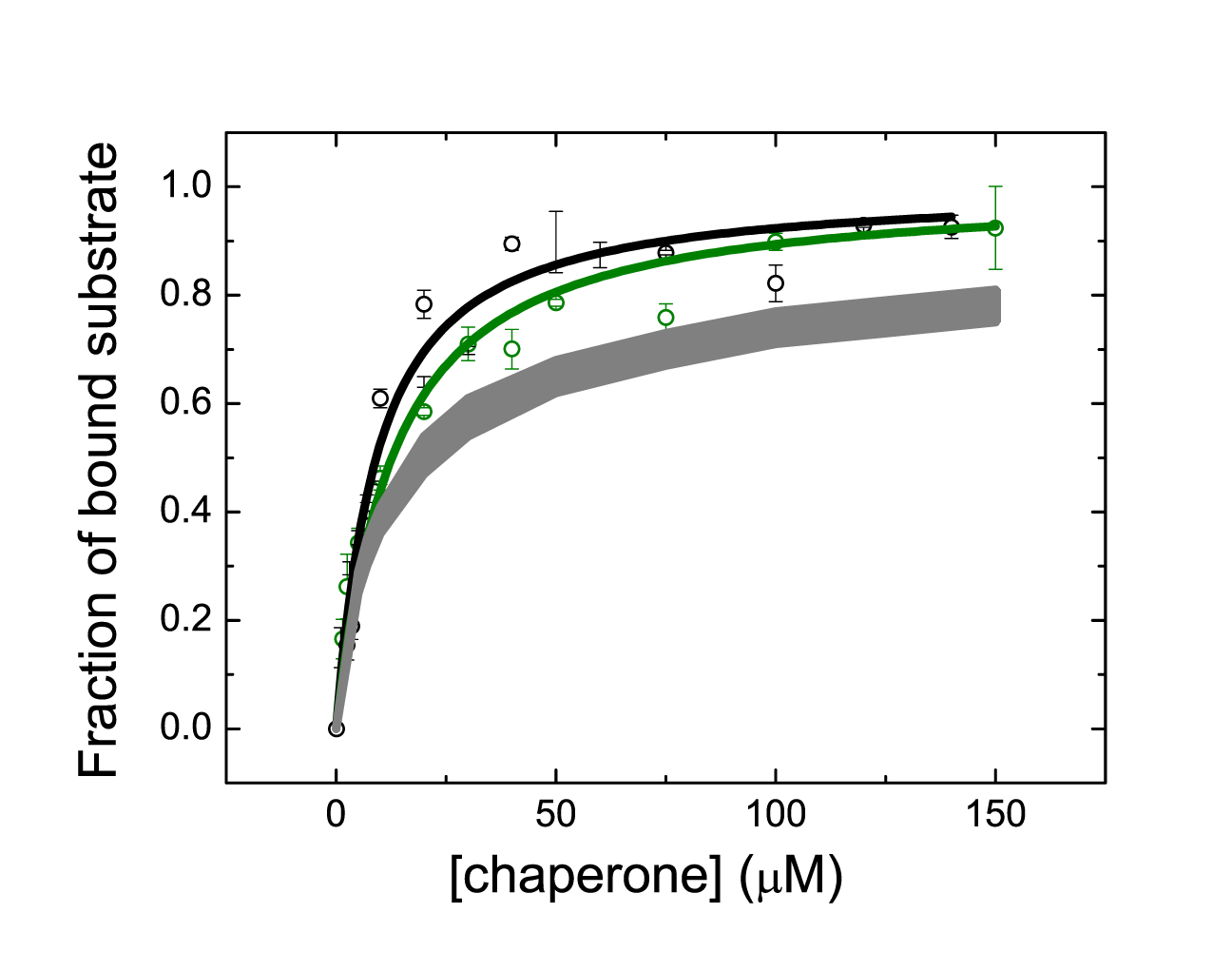

Supplement: Figure S11 — The experimental titration curve of ΔLSBD641 is also shown as reference (black circles and line). The theoretical curves were simulated assuming a competitive model (competition between the linker and the NR peptide for the same chaperone region), solving the law of mass action equation for the equilibrium 2E +S + I (-) ES + EI, where E represents SBD641, S represents the NR peptide and I represents the linker. The total concentrations of species used in the simulation were the same as those used in the experimental titration: [S] = 2µM, [E] = [I] = 0-150 μM. The dissociation constant for the interaction of the NR peptide with the chaperone (Kd(ES) = 8 µM) was obtained from the experimental titration of ΔLSBD641 (variant without linker), and the dissociation constant for the interaction of the linker with SBD in SBD641 was estimated from Table 1 (Kd(EI) = 5-10 µM). (TIF) [file pone.0067961.s012.tif]
